# Elucidating the Structural and Electronic Effects of Ni and Mn Cationic Incorporation on CoOOH for Efficient Benzyl Alcohol Electrooxidation

**DOI:** 10.1021/acsaem.5c04095

**Published:** 2026-04-06

**Authors:** Emmanuel Aransiola, Sahanaz Parvin, Mohamed Ammar, Rachel Smith, Lihua Zhang, Nishu Devi, Barbara R. Evans, Juliane Weber, Jonas Baltrusaitis

**Affiliations:** † Department of Chemical and Biomolecular Engineering, 1687 Lehigh University, 111 Research Dr., Bethlehem, Pennsylvania 18015, United States; ‡ Brookhaven National Laboratory, Center for Functional Nanomaterials, Bldg. 735, Upton, New York 11973-5000, United States; § Oak Ridge National Laboratory, Chemical Sciences Division, Oak Ridge, Tennessee 37831, United States

**Keywords:** Electrooxidation, benzyl alcohol, electrocatalysis, impurities, cobalt oxyhydroxide

## Abstract

Transition-metal oxyhydroxides such as CoOOH are promising low-cost electrocatalysts for the selective electrooxidation of organic molecules, yet the influence of ubiquitous transition-metal impurities on their performance and durability remains poorly understood. Here, we experimentally probed the individual and synergistic electrochemical and structural effects of Ni and Mn incorporations into model CoOOH electrocatalysts toward an efficient benzyl alcohol oxidation reaction (BAOR). Comprehensive electrochemical, microscopic, and spectroscopic analyses reveal that Ni incorporation enhances charge-transfer kinetics and overall activity through the formation of catalytically active Ni^3+^ sites, whereas Mn exhibited a more complex but interesting role. At the early stages of operation, Mn^4+^ acts as a stabilizing surface layer that mitigates catalyst degradation but partially blocks Co sites before they undergo gradual leaching. The concurrent incorporation of both Ni and Mn yields a trimetallic 2NMC@NF electrocatalyst that integrates the activity benefits of Ni with the stability conferred by Mn, achieving 92.9% benzyl alcohol conversion and 91.4% Faradaic efficiency after 24 h at 1.5 V vs RHE. These findings elucidate how trace Ni and Mn impurities, often introduced from electrolytes or external sources, can modulate the lattice and electronic structure of CoOOH, offering a design strategy for enhancing both activity and long-term stability in electrocatalytic organic oxidation.

## Graphical abstract

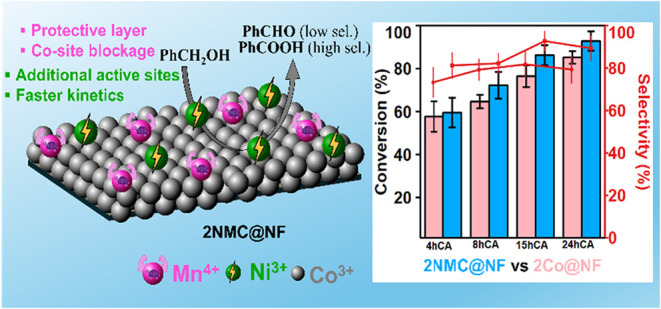

## Introduction

Green hydrogen (H_2_) is of great interest as a sustainable alternative to fossil fuels due to its high gravimetric energy density and carbon dioxide (CO_2_)-free emissions.^1–3^ Hence, developing sustainable and efficient methods for green hydrogen production is of the utmost importance. Electrocatalytic water splitting provides a green and sustainable approach to H_2_ synthesis. An intrinsic limitation is the significant energy requirement of 1.23 V at 25 °C vs RHE and extra potential (overpotential) for the anodic oxygen evolution reaction (OER), stemming from the sluggish kinetics accumulated from four electron–proton coupled reactions.^4,5^ Noble-metal-based electrocatalysts (e.g., Ru and Ir) and their oxides (e.g., RuO_2_ and IrO_2_) have been identified as suitable electrocatalysts for lowering the overpotential for the OER. However, they are limited by prohibitively high cost, limited availability, and durability during prolonged electrochemical reactions.^6,7^ Therefore, replacing the OER from water with a less energy-intensive anodic reaction while also transitioning into more abundant catalyst materials is imperative for feasible large-scale electrocatalytic H_2_ production.

In the past few years, electrocatalytic oxidation of small organic molecules obtained from renewable biomass sources such as alcohols,^8–10^ urea,^11,12^ amines,^13,14^ and aldehydes^15,16^ which are naturally present in water to their corresponding oxidation products, has received a lot of attention. This is mainly due to their significantly lower thermodynamic potential compared to OER. This strategy can efficiently produce H_2_ at the cathode at a relatively low cell voltage, coupled with the synthesis of value-added chemicals at the anode under ambient conditions.^17,18^ In recent years, benzyl alcohol oxidation reaction (BAOR) has gained significant interest not only because of the lower activation energy barrier but also due to the wide application of their oxidation products (e.g., benzaldehyde or benzoic acid) obtained at the anode.^19,20^ Transition-metal oxyhydroxides exhibit excellent activity toward alkaline electrocatalytic anodic oxidation as a result of their abundant active sites on the surfaces and edges, surface reconstruction ability, and tunable electronic structures.^22,23^ Due to their optimal OH–M^2+^δ bond strength based on the Sabatier principle, Co-based catalysts have been extensively used for different electrocatalytic anodic reactions such as OER,^21,22^ hydroxymethylfurfural (HMF) electrooxidation,^23^ and alcohol electrooxidation,^10^ making it suitable for BAOR.

The incorporation of cationic impurities into transition-metal oxyhydroxide catalysts, such as NiOOH and CoOOH, can alter their electrochemical performance. Common sources of cationic impurities stem from precursors used in electrocatalyst synthesis or the electrolyte.^24–26^ Boettcher et al. noted that trace iron (Fe) impurities at ppb levels from commercial KOH and NaOH used as electrolytes significantly improved the OER activity of NiOOH owing to a partial-charge-transfer effect.^27,26^ The increase in the intrinsic catalytic property was due to the Ni cations being replaced by Fe^3+^ as the active site for OER.^25^ A study by Burke et al. demonstrated that CoOOH can absorb Fe impurities from unpurified electrolytes, leading to a significant increase in intrinsic OER activity. The catalyst activity can be enhanced by up to 100-fold when sufficient Fe is incorporated, particularly in compositions where the Fe content reaches *x* ≈ 0.6–0.7 in Co_1–*x*_Fe_*x*_(OOH).^28^ They also noted that, depending on the amount of Fe incorporation, the stability of the catalyst changes. A related study examined the incorporation of La, Mn, Ce, and Ti cations into NiOOH during electrochemical synthesis to probe their impact on OER activity in 1 M Fe-free KOH. Among these, only Ce incorporation resulted in a significant enhancement, transiently increasing NiO_*x*_H_*y*_ activity by about 10-fold before the decrease in performance due to Ce segregation into CeO_2_.^29^ In contrast, Ti incorporation decreased the level of the OER activity, while La provided only a modest improvement at low loading and caused activity loss at higher concentrations.

Studies addressing the structural dynamics during partial electrooxidation of organic moieties are still limited. In a recent work, Yongfang and co-workers systematically introduced Cu^2+^, Ni^2+^, Fe^3+^, Fe^2+^, Co^2+^, Mn^2+^, Zn^2+^, and Ce^3+^ into the electrolyte and evaluated their impact on HMF and glycerol electrooxidation.^30^ They found that Cu^2+^ most strongly promoted these oxidations via *in-situ* formation of Cu­(OH)_2_/CuOOH species, while Ni^2+^ and Fe^3+^ gave moderate enhancement, and Co^2+^/Mn^2+^ mainly boosted water oxidation with only minor effects on HMF and glycerol. In this work, CoOOH electrocatalysts on nickel foam were synthesized in the presence of Ni and Mn ions to obtain model interfaces that potentially represent electrochemically cycled CoOOH in complex trace contaminant-containing electrolyte systems. The activity and stability of Ni- and Mn-modified CoOOH were evaluated electrochemically and by XPS, PXRD, and SEM, and benchmarked against pristine CoOOH (2Co@NF). It was demonstrated that Ni incorporation in CoOOH (2NC@NF) enhanced BAOR performance by improving Co^3+^ active sites formation and introducing additional Ni^3+^ active sites, which together facilitated faster charge-transfer and electrocatalytic kinetics. In contrast, Mn incorporation into CoOOH (2MC@NF) slightly decreased BAOR activity but reduced Co active site loss over time, resulting in more sustained performance over 24 h of CA. Co-incorporation of Ni and Mn (2NMC@NF) delivered a synergistic balance of Ni-driven activity enhancement and Mn-induced stability to produce a superior overall catalyst.

## Experimental Section

### Materials

Porous Ni foam (NF) (10–130PPI, 1 mm thickness from Xiamen Tmax Battery Equipment Limited), cobalt­(II) nitrate hexahydrate (Co­(NO_3_)_2_·6H_2_O; 97.7%, Thermo Scientific), nickel­(II) nitrate hexahydrate (Ni­(NO_3_)_2_·6H_2_O; Fisher chemicals, certified crystalline), manganese­(II) nitrate tetrahydrate (Mn­(NO_3_)_2_·4H_2_O; Thermo Scientific, 98%), ammonium chloride (NH_4_Cl; Sigma-Aldrich, ≥99.5%), hydrochloric acid (HCl; Sigma-Aldrich, 37%w/w), potassium hydroxide pellets (KOH; Sigma-Aldrich, ≥99%), isopropanol (C_3_H_7_OH; Merck, ≥99.9%), Toray carbon fiber paper (CFP; ThermoScientific), benzyl alcohol (C_6_H_5_CH_2_OH; Fisher chemical), and benzoic acid (C_6_H_5_COOH; Thermo Scientific, 99.5% min), benzaldehyde (C_6_H_5_CHO; Sigma-Aldrich, ≥99%), Acetonitrile (CH_3_CN; Sigma-Aldrich, ≥99.9%), phosphoric acid (H_3_PO_4_; Sigma-Aldrich, 85 wt % in H_2_O). All materials mentioned were used without further purification except for NF.

### Synthesis of Cobalt Oxyhydroxide with Ni and Mn Impurities on Nickel Foam

All model CoOOH-based electrocatalysts were synthesized via a two-step electrochemical process on a NF current collector. Prior to electrodeposition, NF substrate (1 cm by 0.6 cm double-sided, 1.2 cm^2^ total area) was flattened to minimize NF reconstruction to Ni­(OH)_2_ and ensure uniform catalyst coverage (Figure S1).^31^ In a typical procedure, Co­(OH)_2_ (denoted as 1Co@NF) was first electrodeposited from an aqueous electrodeposition bath containing 100 mM Co­(NO_3_)_2_·6H_2_O and 0.03 M NH_4_Cl. NH_4_Cl was used to improve the ionic conductivity of the solution. Electrodeposition was performed using a standard three-electrode setup comprising NF as the working electrode, Ag/AgCl (saturated KCl) as the reference electrode, and nonfluorinated carbon fiber paper (CFP, 4.5 cm^2^) as the counter electrode.

1Co@NF was first electrodeposited at −0.8, −1.0, and −1.2 V vs Hg/HgO. All electrodeposited catalysts were subsequently oxidized in 1 M KOH via cyclic voltammetry (CV) (0.9–1.6 V vs RHE, 30 cycles, 10 mV s^–1^) to form CoOOH (2Co@NF). As the electrocatalyst deposited at −1.2 V vs Ag/AgCl/Cl^–^ (saturated KCl) showed the lowest overpotential in the presence of 0.1 M benzyl alcohol (Figure S2a), electrodeposition time was further varied (8, 12, and 16 min) at a constant potential of −1.2 V vs Ag/AgCl/Cl^–^ (saturated KCl). The electrocatalytic activity of the electrocatalysts deposited for 12 min showed the lowest overpotential at 10 mA cm^–2^ (Figure S2b); hence, these conditions were selected as the standard conditions for all syntheses. After oxidation, the resulting 2Co@NF electrocatalyst was rinsed with deionized water, air-dried, and stored in airtight containers.

Manganese-incorporated CoOOH (2MC@NF), nickel-incorporated CoOOH (2NC@NF), and both nickel- and manganese-incorporated CoOOH (2NMC@NF) were subsequently synthesized at −1.2 V versus Ag/AgCl/Cl^–^ for 12 min and oxidized by the same 30-cycle CV protocol. Prior to fabrication of optimized 2NMC@NF, different concentrations of Mn and Ni precursors were tested for fabrication of optimized 2NMC@NF. To maintain the total metal concentration at 100 mM and to ensure that Co remained the primary metal component, the Co precursor concentration was fixed at 62.5 mM, and the Ni and Mn precursor concentrations were systematically varied. Specifically, a series of electrodepositing baths with different Ni/Mn ratios were prepared (e.g., 10/27.5, 12.5/25, 15/22.5, 17.5/20, and 20/17.5 mM). The Ni and Mn precursors were varied in a complementary manner from high-Mn/low-Ni to high-Ni/low-Mn compositions to construct a monotonic compositional gradient, which allowed efficient identification of the optimal intermediate Ni/Mn ratio within a narrow number of experiments. The resulting electrocatalysts were then evaluated for BAOR activity. Figure S2c summarizes the current densities at a constant potential of 1.4 V vs RHE obtained for different Ni/Mn precursor concentrations toward BAOR, with the highest current density achieved using 12.5 mM Ni, 25 mM Mn, and 62.5 mM Co. These optimized trimetallic precursor concentrations were therefore used for all 2NMC@NF syntheses. The detailed electrocatalyst nomenclature is provided in Table S1.

The optimized loading amount of the as-synthesized 2Co@NF and 2NMC@NF was estimated to be 1.5 and 1.35 mg cm^–2^, respectively, using the difference in the weight before and after electrodeposition on an NF and CV oxidation.

### Physicochemical Characterization

X-ray diffraction patterns were collected on an Empyrean X’pert PRO diffractometer using Cu Kα = 1.54059 Å radiation, with a scan range of 5–80° and a scan step of 5° min^–1^. The morphology of the prepared sample was analyzed by field emission scanning electron microscopy (FESEM, Hitachi S-4300SE/N Electron microscope) using an accelerating voltage of 5 kV and gun brightness of 3. SEM-EDS Elemental distribution maps were collected by energy dispersive spectrometry (EDS, 30 mm^2^ EDAX Octane Elect Plus Octane Elect Plus Silicon Drift Detector) using an accelerating voltage of 15 kV and gun brightness of 5. High-angle annular dark field scanning TEM (HAADF-STEM) mapping was obtained with a Thermo Fisher Talos 200X, a 200 keV high-resolution analytical scanning/transmission electron microscope equipped with a four-quadrant energy dispersive X-ray spectrometer for elemental and compositional mapping. For HAADF-STEM analysis, the electrocatalyst deposited on Ni foam was dispersed in isopropanol and sonicated for 10 min. The resulting suspension was then diluted and drop-cast onto a Cu lacey carbon film grid.

SPECS XPS spectrometer equipped with a μ-FOCUS 600 X-ray monochromator operating in UHV mode was used to acquire spectra. Al Kα radiation was used with an X-ray beam energy of 1486.7 eV and a power of 100 W. A PHOIBOS 1D-DLD hemispherical analyzer (0.85 eV energy resolution) was used to acquire the spectra. Survey spectra were acquired by using a pass energy of 100 eV, a step size of 1 eV, and a dwell time of 100 ms. High-resolution scans were acquired by using a pass energy of 20 eV, a step size of 0.1 eV, and a dwell time of 1 s. Scofield relative sensitivity factors (RSF)^32^ were used in quantification together with the instrument-measured transmission function and effective attenuation length correction (EAL).^33^ CasaXPS v2.3.6rev1.0Q was employed for all data processing tasks.^34^ The inelastically scattered background was subtracted using the Shirley background.^35^ The spectra were calibrated using a C 1s binding energy of 285.0 eV.

Metal content of aqueous solutions was determined using Inductively Coupled Plasma-Optical Emission Spectroscopy (ICP-OES) in radial mode by using an iCAP 7400 spectrometer (Thermofisher Scientific) equipped with a prepFast M5X intelligent dilution automatic sample loader (Elemental Scientific, Omaha, Nebraska). Cobalt emission was measured at 228.616, 238.892, 237.862, 230.786, and 231.160 nm; nickel was measured at 221.647, 231.604, 232.003, and 252.454 nm; and manganese was measured at 260.569, 257.610, and 403.307 nm. 2% nitric acid (J.T. Baker Ultrapure II) in distilled water purified with a Milli-Q purification system (Millipore) was the diluent and carrier solvent. The internal standard was 5 ppm of yttrium. Standard stock solutions containing 50 ppm of cobalt, 50 ppm of nickel, and 5 ppm of manganese were prepared from commercial single elemental standards for these metals (cobalt and manganese from High Purity Standards, Charleston, South Carolina, USA; Trace-Cert nickel from Sigma-Aldrich, St. Louis, MO, USA). Automatic dilution by the sample loader was used to generate 7-point standard curves. The limit of Detection (LOD) was 0.5 ppm according to the manufacturer. Experimental solutions were filtered through syringe filters with a 0.45 μm pore size. Since the filtered electrolyte solutions contained 1 M KOH, they were diluted 20-fold with 2% HNO_3_, giving a 2.8-fold molar ratio of HNO_3_ to KOH in the samples to ensure acidification for ICP-OES. Solutions from the dissolution of electrode materials in nitric acid were diluted 4-fold with purified distilled water to bring final HNO_3_ concentrations to 2%. The standard stock solution was analyzed as a Quality Control Check at the start of the run to check accuracy. The Qtegra instrument software calculated the original concentrations in parts per million for each sample based on the dilution factors that were entered into the method.

*Ex-situ* Raman measurements were performed using a WITec alpha300R confocal Raman microscope equipped with a 532 nm laser, ZEISS LD ACHROPLAN 50x/0.75 HD objective, and G2:600 g mm^−1^ grating. The spectral range was 0–4000 cm^−1^ with the spectra center at 2200 cm^−1^. The laser intensity in the sample was ∼5% to minimize any laser-induced heating effects. Spectra were acquired using 10 consecutive scans, with 5 s exposure time per scan. For good reproducibility of the obtained spectra, we calibrated the energy shift using the 520 ± 1 cm^−1^ peak of silicon wafer.

### Quantitative Analysis of Liquid Products

The electrochemical conversion of benzyl alcohol to corresponding products (aldehyde and acid) was analyzed and quantified by high-performance liquid chromatography (HPLC; Agilent 1200 Series) equipped with a C18 column of 4.6 mm × 150 mm (Restek Pinnacle II, 5 μm Particle Size) and detected by a 210 nm UV detector. The column oven temperature was maintained at 40 °C. A mixture of water and acetonitrile in a 70:30 volume ratio with 0.2 M phosphoric acid was used as the mobile phase. The mobile phase flow rate was maintained at 1 mL min^–1^. The aliquots were prepared by diluting 20 μL of electrolyte with 530 μL of deionized water. Calibration curves for benzyl alcohol, benzaldehyde, and benzoic acid were obtained by taking HPLC measurements of 1 M KOH + the corresponding standard solution. The concentrations of the standard solutions varied from 5 to 300 mM. Predetermined response factors from calibrations of benzyl alcohol, benzaldehyde, and benzoic acid were used to determine benzyl alcohol conversion and catalytic activity.

For product analysis during BAOR, chronoamperometry was performed at 1.5 V vs RHE in 30 mL of 1.0 M KOH containing 0.1 M benzyl alcohol for 24 h using 2NMC@NF or 2Co@NF as the working electrode. Electrolyte aliquots (20 μL) were withdrawn after 1–8 h (every hour) and at 12, 15, and 24 h for HPLC analysis.

### Electrochemical Measurements

All electrochemical measurements during BAOR were performed in 1 M KOH + 0.1 M benzyl alcohol, pH 14, at room temperature on a Pine Research Wavedriver 200 electrochemical workstation. The electrochemical tests were performed in a standard three-electrode system in a membrane-free glass beaker, using the as-synthesized electrocatalyst fabricated on Ni foam, Hg/HgO/OH^–^ (1 M NaOH) and 1.5 cm × 1.5 cm CFP as working, reference, and counter electrode, respectively. Additionally, OER measurements were carried out in a 1 M KOH electrolyte, using a similar three-electrode setup. LSV polarizations of the electrocatalysts were acquired at a low scan rate of 1 mV s^–1^ in backward scan mode to avoid current contribution from double layer capacitance (*C*_dl_), and metal oxidation, respectively. The working electrode was stabilized and cleaned by 50 cyclic voltammetry (CV) measurements at a scan rate of 50 mV s^–1^. The current density was calculated by normalizing the current with respect to the geometric area of both sides of the working electrode. Total charge (*Q*, mC cm^–2^) was calculated from CVs (*E*–*i* data) by converting potential to time using the scan rate, ν, (*t* = *E*/ν), followed by integration of the anodic current density region using Pine Research AfterMath v2.214955. All of the potential was 85% *iR*-corrected and converted to the reversible hydrogen electrode (RHE) scale using the Nernst (eq 1) as below unless specified.

1 $$E_{\mathrm{RHE}}=E_{\mathrm{Hg}/\mathrm{HgO}}+0.098+0.059\times \mathrm{pH}$$

where *E*_RHE_ = potential vs RHE, *E*_Hg/HgO_ = potential vs counter electrode (Hg/HgO). All potentials are thus referred to the RHE scale unless stated differently. *C*_dl_ of all the electrocatalysts was estimated from 20 to 100 mV s^–1^ CV scans in the non-Faradaic region with no noncapacitive current (potential range between 0.91 and 0.95 V vs RHE). The Δ*J* (mA cm^–2^) estimated by averaging the cathodic and anodic current densities at a potential of 0.93 V versus the RHE was plotted against the scan rates. This relates to the linear behavior characteristic of an ideal capacitor. The slope of the fitted line corresponds to the value of *C*_dl_, which was compared between the electrodes.

Tafel slopes were calculated from LSV curves using the Tafel equation: η = *a* + *b* log|*j*|,^36^ where η represents the overpotential, *b* is the Tafel slope, *a* is a constant, and *j* is the current density. The conversion tests for BAOR were performed by the CA method at 1.5 V vs RHE for 24 h. 20 μL of the electrolyte was collected at intervals of 2 h for HPLC measurements.

Temperature-dependent kinetic study experiments were carried to gain deeper insight into the kinetics of 2Co@NF and 2NMC@NF electrocatalysts, LSV scans were conducted in 1 M KOH + 0.1 M benzyl alcohol across the temperature range of 303 K–353 K, with a 10 K increment and continuous stirring at 700 rpm in a customized and sealed three-electrode cell. To limit pressure build-up and potential concentration gradients of benzyl alcohol at higher temperatures due to atmospheric interactions, a small vent on the sealing cap was periodically opened. The Arrhenius plot was evaluated by taking the logarithm of the current densities and the inverse of the corresponding temperature from LSV plots at a potential of 1.38 V vs RHE.

Benzyl alcohol conversion, Faradaic efficiency (FE), selectivity, and yield for benzoic acid formation from BAORs were calculated based on their corresponding electron transfer per molecule oxidation using ([Disp-formula eq2]–[Disp-formula eq5])­

2a $$\%\mathrm{conversion}=\frac{\mathrm{mol}\;\mathrm{of}\;\mathrm{reacted}\;\mathrm{benzyl}\;\mathrm{alcohol}}{\mathrm{mol}\;\mathrm{of}\;\mathrm{initial}\;\mathrm{benzyl}\;\mathrm{alcohol}}\times 100\%$$

2b $$\%\mathrm{FE}=\frac{\mathrm{mole}\;\mathrm{of}\;\mathrm{benzonic}\;\mathrm{acid}\;\mathrm{formed}\times 4F}{\mathrm{total}\;\mathrm{charge}\;\mathrm{passed}}\times 100\%$$

2c $$\%\mathrm{selectivity}=\frac{\mathrm{mole}\;\mathrm{of}\;\mathrm{benzonic}\;\mathrm{acid}\;\mathrm{formed}}{\mathrm{mol}\;\mathrm{of}\;\mathrm{reacted}\;\mathrm{benzyl}\;\mathrm{alcohol}}$$

2d $$\%\mathrm{yield}=\frac{\mathrm{mol}\;\mathrm{of}\;\mathrm{benzoic}\;\mathrm{acid}\;\mathrm{formed}}{\mathrm{mol}\;\mathrm{of}\;\mathrm{initial}\;\mathrm{benzyl}\;\mathrm{alcohol}}\times 100\%$$

where the total charge passed is obtained from the integral of current (*I*, A) and its corresponding time (*t*, s), using the CA plot. *F* is the Faraday constant (96,500 C mol^–1^).

## Results and Discussion

### Electrochemical Synthesis and Physical Characterization of 2Co@NF and 2NMC@NF

A schematic illustration of 2NMC@NF electrocatalyst fabrication as detailed in the Experimental Section is shown in Figure 1a. The resulting electrocatalysts change color from green to black, characteristic of cobalt oxyhydroxide. Figure 1b shows the CV oxidation profiles of all electrocatalysts after 30 activation cycles of CV. The peaks observed at 1.208 and 0.968 V in 2Co@NF correspond to oxidation of Co^2+^ to Co^3+^ and subsequent reduction to Co^2+^ during the forward and backward scan, respectively.^37,38^ The absence of additional redox features attributable to Ni or Mn in 2NC@NF, 2MC@NF, and 2NMC@NF indicated that these cations are completely incorporated into the CoOOH lattice rather than forming separate phases. However, Mn incorporation induced a 30 mV negative shift in the Co^2+^/Co^3+^ oxidation potential, while Ni incorporation caused a 40 mV positive shift. In alkaline media, the Ni^2+^/Ni^3+^ redox transition in Ni-containing oxyhydroxides generally occurs at more positive potentials than the Co^2+^/Co^3+^ couple in Co-based oxyhydroxides, therefore, the observed positive shift is consistent with Ni incorporation effects.^39,40^ A 2 mV negative shift in 2MNC@NF signified both Ni and Mn incorporation.

**Figure 1:**
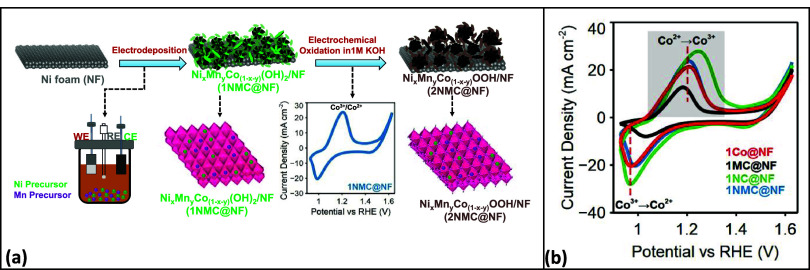
(a) Schematic illustration of 2NMC@NF synthesis by electrodeposition to fabricate Ni and Mn incorporated Co­(OH)_2_ (1NMC@NF), followed by CV oxidation in 1 M KOH to form 2NMC@NF. (b) Comparison of electrochemical CV oxidations of the as-electrodeposited 1Co@NF, 1NC@NF, 1MC@NF, and 1NMC@NF films at 10 mV s^–1^ in 1 M KOH. WE, RE, and CE are the working, reference, and counter electrodes, respectively. The gray shaded area in panel (b) corresponds to Co^2+/3+^ oxidation peak area in Table 1.

Table 1 summarizes the oxidation and reduction potentials and integrated peak areas for all electrocatalysts. The integrated peak area associated with Co^2+^/Co^3+^ revealed the formation of higher Co^3+^ active site density in 1NC@NF (132.7 mC) and 1NMC@NF (121.5 mC) that can translate to higher electrochemical activity. The lower charge for 1MC@NF (69.7 mC) suggests possible blocking of Co sites by surface Mn-rich species.

**Table 1:** Co^2+/3+^ Oxidation and Reduction Potentials and Oxidation Peak Areas for All Electrodeposited Electrocatalysts during CV Oxidation in 1 M KOH^a^

electrocatalysts	*E*_Co^2+^→Co^3+^_ (V vs RHE)	oxidation peak area (mC)	*E*_Co^3+^→Co^2+^_ (V vs RHE)
1Co@NF	1.208 ± 0.022	96.82 ± 24.58	0.968 ± 0.17
1MC@NF	1.178 ± 0.031	69.74 ± 17.01	1.027 ± 0.23
1NC@NF	1.248 ± 0.012	132.74 ± 27.89	0.964 ± 0.14
1NMC@NF	1.206 ± 0.009	121.52 ± 21.43	0.988 ± 0.01

a mC cm^–2^ was converted to mC by multiplying by the geometric area (1.2 cm^2^). (Errors calculated from 3 independent readings).

FESEM morphological analysis revealed that electrodeposited 1Co@NF (Figure S3a) formed dense 3D microflower assemblies of tightly packed ultrathin nanosheets.^41^ Similar morphology is observed in 1NMC@NF (Figures S3b). Upon electrochemical oxidation of 1Co@NF to 2Co@NF, more open sheet-based flowers with increased edge exposure (Figure 2a), consistent with prior reports, were observed.^38,42^ Mn incorporation in 2MC@NF (Figure S3c) yielded nanosheets that are relatively compact and platelike, forming dense flower-like aggregates with thicker sheets compared to pristine CoOOH, suggesting that Mn incorporation promoted slightly thicker, more stacked sheets. In contrast, 2NC@NF (Figure S3d) exhibited loosely packed and more crumpled plates, with protruding edges suggesting that Ni incorporation promoted a more open, defect-rich architecture with higher edge density and void space between sheets, enhancing electrolyte access and the number of undercoordinated active sites. The ternary 2NMC@NF (Figure 2b) exhibited densely packed microflowers enriched with needle-like protrusions, demonstrating that metal incorporations systematically modulated nanosheet thickness, packing density, and edge/defect features within the retained CoOOH nanoarchitecture.

**Figure 2:**
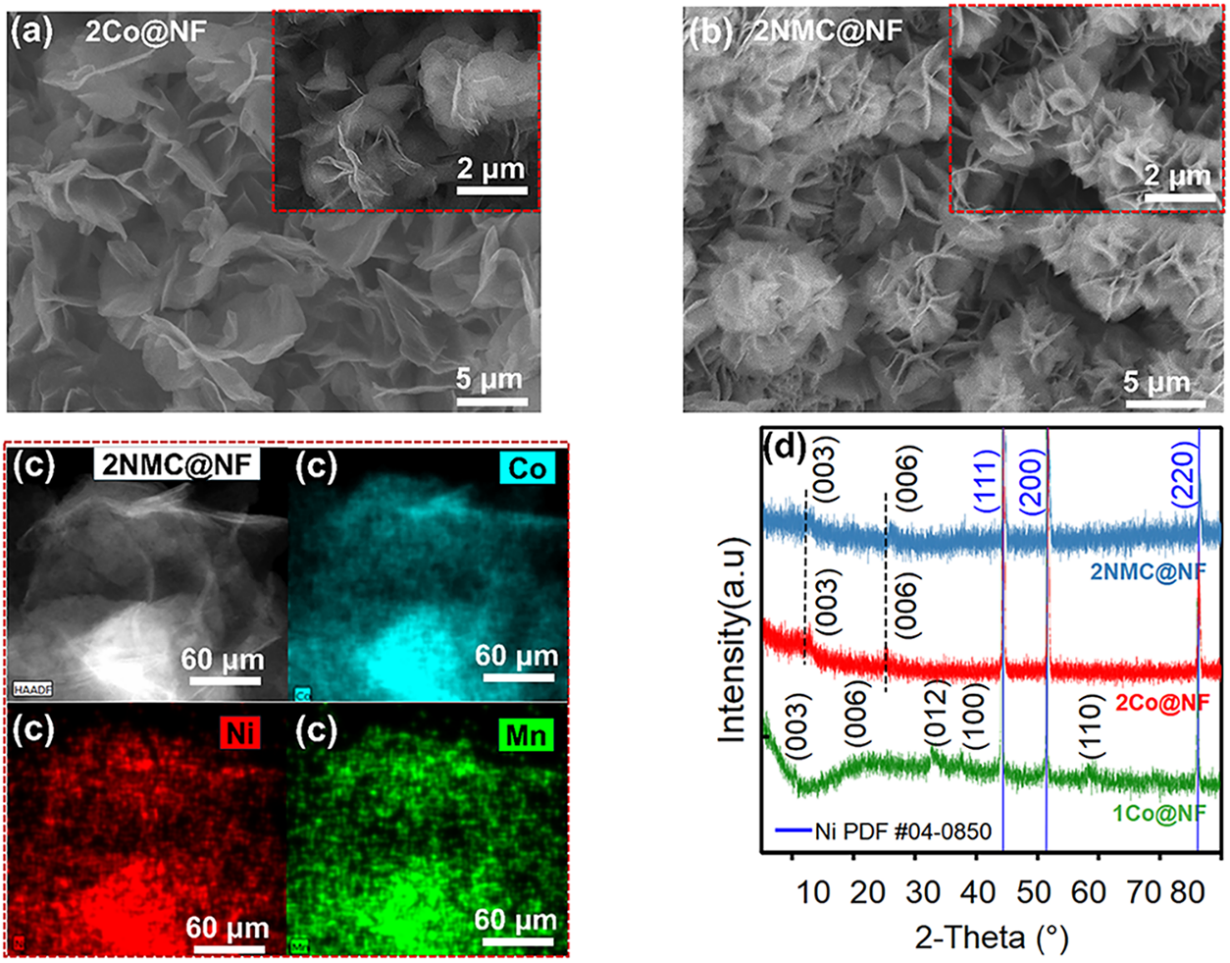
FESEM images of (a) 2Co@NF, (b) 2NMC@NF, (c) STEM elemental mapping of Co, Ni, and Mn in 2NMC@NF, and (d) PXRD patterns of 1Co@NF, 2Co@NF, 2NMC@NF. The sharp reflection peaks at 2θ = 44.5°, 51.8°, and 76.4° corresponding to (111), (200), and (220) planes, due to the NF substrate (JCPDS card no. 04–0850).^43^

STEM elemental mapping (Figure 2c) was performed to verify Ni and Mn incorporation in 2NMC@NF, while SEM-coupled EDS and corresponding EDX spectra (Figure S4a,c) were used to quantify the elemental composition. STEM elemental mapping confirmed the homogeneous distribution of these elements across the nanosheet structure. SEM-EDS analysis further revealed a Ni/Mn/Co atomic ratio of ∼0.06:0.21:1 in 2NMC@NF (Figure S4b), consistent with the uniform metal distribution in STEM maps (Figure 2c).

Powder X-ray diffraction (PXRD) analysis of the precursor 1Co@NF can be indexed to poorly crystalline α-Co­(OH)_2_ phase, with characteristic broadened but discernible (003), (006), (012), (100), and (110) reflections at 10.2°, 22.1°, 33.3°, 36.4°, and 59.1° respectively (JCPDS no. 46–0605).^19,44,45^ Upon electrochemical oxidation, a 2Co@NF γ-CoOOH phase is observed, evidenced by the appearance of (003) and (006) reflections at approximately 12.7° and 26.3°, respectively. The high noise ratio and weak diffraction peaks observed for all electrocatalysts indicate a low degree of crystallinity characteristic of electrodeposited catalysts.^46^ After Ni and Mn incorporation (2NMC@NF), the γ-CoOOH structure was largely preserved, with no additional diffraction features attributable to secondary Ni- or Mn-phases. Instead, positive 2θ shifts of 0.2° and 0.4° in the respective (003) and (006) planes of γ-CoOOH due to the difference in ionic radii of Co, Mn, and Ni.^47^ Raman spectroscopy, which is well suited for poorly crystalline materials, was used to verify the structures of the as-synthesized hydroxide and oxyhydroxide electrocatalysts (Figure S5a,b). In as-synthesized hydroxides, Figure S5a, the peaks at 448 and 518 cm^–1^ are attributed to O–Co–O bending vibration and symmetric stretching mode of CoO (A_2g_), respectively.^48^ For Ni and Mn incorporation, a slight shift is observed in A_2g_. For 2Co@NF, Figure S5b, two peaks at 501 and 585 cm^–1^ observed for 2Co@NF were assigned to E_g_ and A_1g_ vibrational modes of Co–O in CoOOH. Ni and Mn incorporation in 2NMC@NF induced a red shift in the A_1g_ mode (501 → 491 cm^–1^) and a blue shift in the E_g_ mode (585 → 605 cm^–1^), indicative of lattice perturbation upon Ni and Mn substitution.^22^

To probe how Ni and Mn incorporation affects the Co chemical environment in the hydroxide precursor, Co 2p XPS spectrum of as-synthesized 1Co@NF and 1NMC@NF (Figure S6). The Co 2p_3/2_ high-resolution (HR) spectrum was peak-fitted using the model and parameters for Co­(OH)_2_ described by Biesinger et al.^49^ As shown in Figure S6, Ni and Mn incorporation in 1NMC@NF caused the Co 2p peak shift by ∼0.4 eV. This is due to the significant electronic interactions between Co, Ni and Mn and changes in the local electronic environment of Co. After electrochemical oxidation in 1 M KOH, the peak-fitted XPS spectra of 2Co@NF, 2NMC@NF, 2NC@NF and 2MC@NF are shown in Figure 3a–d. The Ni 2p_3/2_ spectra of 2NC@NF indicated the presence of both Ni^2+^ and Ni^3+^; however, based on the average binding energies and the asymmetric peaks at 856.1 eV (Ni 2p_3/2_) and 873.9 eV (Ni 2p_1/2_), along with their corresponding satellite peaks at 861.7 and 880.2 eV, respectively, Ni exists majorly in the +3 state (Figure 3a).^50–52^ No obvious chemical shift for Ni 2p was observed in 2NMC@NF as compared with 2NC@NF electrocatalysts. A small shoulder peak in the XPS spectra of 2NMC@NF at around the binding energy of 852.5 eV shows the presence of the Ni^0^ oxidation state, which likely arises from the NF substrate. The Co 2p_3/2_ peak fit (Figure 3b) matches closely with that of Co spinel oxide (Co_3_O_4_), confirming the coexistence of Co^2+^ and Co^3+^ in all electrocatalysts.^49^ Additionally, the spin–orbit splitting of approximately 15 eV for Co 2p is consistent with the coexistence of Co^2+^ and Co^3+^ oxidation states (Figure 3b).^53–57^ However, the Co 2p_3/2_ main peak around ∼780.0 eV and relatively weaker shakeup satellites indicated that Co^3+^ is the predominant oxidation state,^49^ consistent with observed γ-CoOOH in bulk PXRD. The shift to higher binding energy remained observable in 2MC@NF and 2NMC@NF when compared to pristine 2Co@NF as Co 2p_3/2_ shifted by 0.45 and 0.52 eV, respectively (Figure 3b). The features at ∼789.9 eV correspond to Co 2p_3/2_ satellites. For Mn 2p XPS in Figure 3c, the absence of satellite peaks at around 648 eV eliminates the possibility of Mn^2+^ in 2NMC@NF.^49,58^ The Mn 2p_3/2_ Manganite (MnOOH) fitting model by Biesinger et al. was used for fitting Mn, which entailed five multiplet-split components.^49^ However, due to the presence of a satellite peak around 665 eV, Mn exists predominantly in the +4 oxidation state (Figure 3c).^59^ Comparing both the 2MC@NF and 2NMC@NF electrocatalysts, no significant shift was observed in the respective binding energies of Mn 2p_1/2_ and Mn 2p_3/2_. However, there is a 0.3 eV decrease in the spin–orbital splitting of 2NMC@NF, possibly caused by electronic modulation induced by Ni incorporation. Since the 665 eV satellite peak remained detected, the mixed +3 and +4 oxidation states are present.

**Figure 3:**
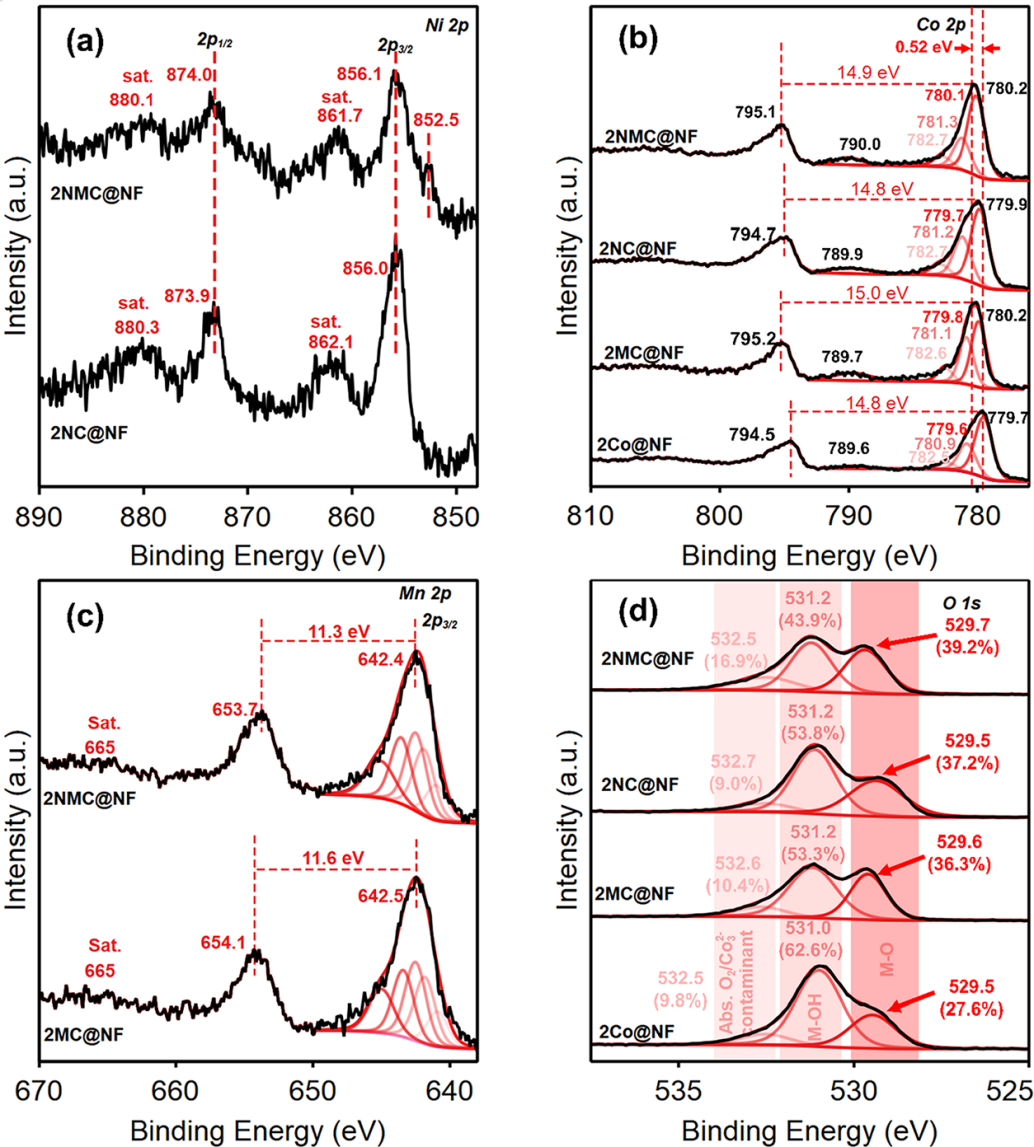
XPS of (a) Ni 2p for as-synthesized 2NC@NF and 2NMC@NF, (b) Co 2p for as-synthesized 2Co@NF, 2MC@NF, 2NC@NF, and 2NMC@NF, and (c) Mn 2p for as-synthesized 2MC@NF and 2NMC@NF, (d) O 1s for as-synthesized 2Co@NF, 2MC@NF, 2NC@NF, and 2NMC@NF.

The complex O 1s peak of 2Co@NF was peak-fitted into three components at 529.5, 531.0, and 532.5 eV, corresponding to lattice oxygen (O^2–^ in M–O), hydroxyl groups (M–OH), and surface-adsorbed oxygen species (O_2_^–^/O_2_^2–^) and/or carbonate contaminants. (Figure 3d), respectively.^22,60^ The peak-fitted O 1s peaks showed that the ratio of the Co–O peak at 529.5 −529.7 eV increased with Ni and Mn incorporation (2NMC@NF > 2NC@NF > 2MC@NF > 2Co@NF), revealing the dynamic surface changes with metal incorporation. Metal oxide bonds (M-O) have been reported to enhance electrocatalytic oxidation reactions, such as OER.^61^ This could play a critical role in the improved performance of 2NC@NF and 2NMC@NF toward BAOR. The increase in the ratio of Co–O to Co–OH from 2Co@NF to 2NMC@NF can be attributed to the modified surface chemistry induced by Ni and/or Mn incorporation.

### Electrochemical Activity and Stability Tests of 2Co@NF, 2NC@NF, 2MC@NF, and 2NMC@NF toward BAOR

The electrocatalytic BAOR activity of the as-synthesized 2Co@NF and 2NMC@NF was tested in 1.0 M KOH + 0.1 M benzyl alcohol (pH = 14.0) electrolyte using a standard three-electrode system. Electrocatalytic performance was compared to that of single-metal-doped CoOOH electrocatalysts (2NC@NF, 2MC@NF), and pristine NF under identical conditions. The potential requirements to reach current densities of 10 and 50 mA cm^–2^ were compared. From the polarization curves (Figure 4a), both 2NMC@NF and 2NC@NF showed similar onset potentials and comparable potentials to reach 10 mA cm^–2^, however, at higher current densities, 2NC@NF slightly outperformed 2NMC@NF. This suggests that while 2NMC@NF maintains competitive activity at moderate currents, 2NC@NF may have an edge in sustaining performance at elevated current densities, likely due to optimized active site exposure and electronic effects induced by Ni incorporation, whereas 2MC@NF shows negligible improvements relative to 2Co@NF. Detailed potential requirements for BAOR onset and to reach 10 and 50 mA cm^–2^ are presented in Figure 4b and Table S2. In fact, at higher current density, 2MC@NF showed a slight decrease in activity compared to 2Co@NF. Previous reports likewise showed no noticeable improvement in CoOOH activity toward OER due to Mn doping^62^ which was attributed to the predominance of electrocatalytically in-active Mn^4+^ species.^63^ Here, the surface of 2MC@NF was enriched in Mn^4+^ via XPS, which will inhibit access to active sites.

**Figure 4:**
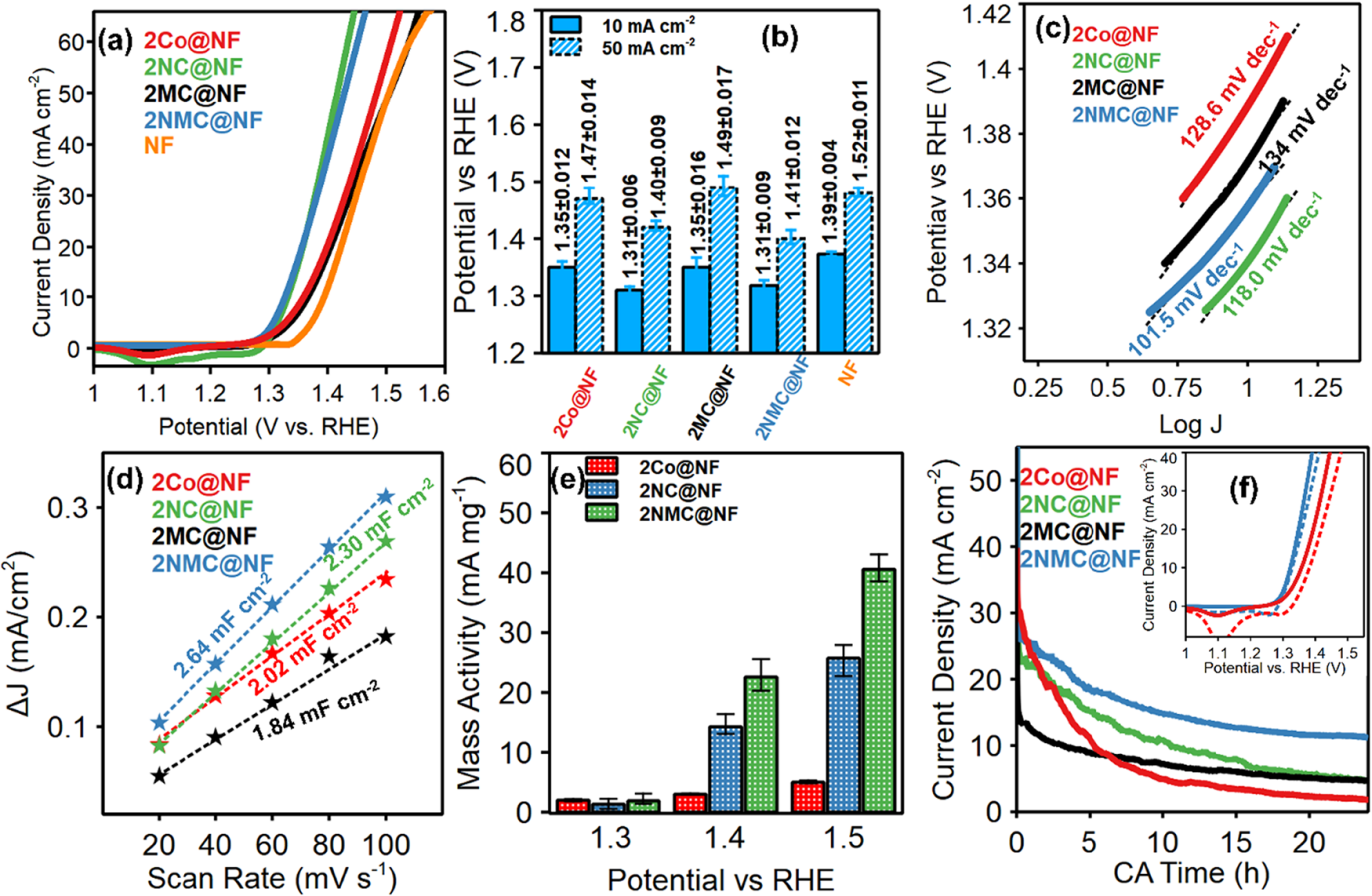
BAOR performance of all electrocatalysts in 1 M KOH + 0.1 M benzyl alcohol. (a) LSV polarization curves for BAOR (*iR*-corrected) for 2Co@NF, 2NC@NF, 2MC@NF, and 2NMC@NF catalysts fabricated on Ni foam and bare Ni foam (backward scan; scan rate of 1 mV s^–1^). (b) Bar plots for overpotentials at 10 and 50 mA cm^–2^. (Error bars computed from 3 different measurements with different electrodes) (c) Tafel plots for 2Co@NF, 2NC@NF, 2MC@NF, and 2NMC@NF (obtained from *iR-*corrected LSV measurement), (d) *C*_dl_ plot for 2Co@NF, 2NC@NF, 2MC@NF, and 2NMC@NF. (e) Mass activity of 2Co@NF, 2NC@NF, and 2NMC@NF catalysts fabricated on Ni foam. (error bars computed from 2 different electrodepositions) and (f) CA plots of 2Co@NF, 2NC@NF, 2MC@NF, and 2NMC@NF at a constant potential of 1.5 V vs RHE (the inset plots show LSV polarization plots before and after 24 h CA. Solid red: 2Co@NF in fresh electrolyte before 24 h CA, dashed red: 2Co@NF in fresh electrolyte after 24 h CA, Solid blue: 2NMC@NF in fresh electrolyte before 24 h CA, dashed blue: 2NMC@NF in the fresh electrolyte after 24 h CA). (All measurements in 1 M KOH + 0.1 M benzyl alcohol).

Tafel plots were derived from the LSV polarization curves for each electrocatalyst to analyze the reaction mechanism and kinetics of BAOR (Figure 4c). 2NMC@NF exhibited the lowest Tafel slope (101.5 mV dec^–1^), revealing faster charge-transfer kinetics and higher electrochemical reaction rates. The highest Tafel slope of 134 mV dec^–1^ for 2MC@NF suggests that BAOR proceeds at a slower rate. Compared to 2Co@NF, 2NC@NF indicates improved kinetics due to an increased number of Co^3+^ active sites and the incorporation of conductive Ni active sites. The difference in Tafel slopes of 2NC@NF and 2NMC@NF despite similar activities is attributed to synergistic Ni–Mn co incorporation, which further optimizes the electronic structure of CoOOH leading to enhanced charge transfer efficiency. Figure 4d shows the *C*_dl_ plots for 2Co@NF, 2NC@NF, 2MC@NF, and 2NMC@NF, obtained from CVs (Figure S7a–d) recorded between 20 and 100 mV s^–1^ at a nonfaradaic potential region (0.91 to 0.96 V vs RHE). For each electrocatalyst, the current density was sampled at 0.93 V vs RHE, and the slope of current density versus scan rate under these nonfaradaic conditions yielded the *C*_dl_ for insights in electrochemically active surface area and the density of accessible surface sites. The highest *C*_dl_ value (2.64 mF cm^–2^) for 2NMC@NF reflected the largest population of electrochemically active sites for charge storage and interfacial reactions among all tested electrocatalysts.

The mass activity of electrocatalysts offers a way to measure the electrocatalyst efficiency and scalability, which are important for industrial-scale electrochemical reactions. Figure 4e shows the mass activity for 2Co@NF, 2NC@NF, and 2NMC@NF derived from LSV polarization plots by normalizing the current density with respect to loading (i.e., the amount of the electrocatalyst electrodeposited on NF). As the potential increases from 1.3 to 1.5 V vs RHE, the mass activity for all three electrocatalyst increases, indicating enhanced catalytic performance at higher potentials. 2NMC@NF demonstrated the highest mass activity, particularly at 1.5 V vs RHE, suggesting enhanced catalytic efficiency at higher potential.

While electrochemical activity is important in analyzing the performance of electrocatalysts, the stability of these electrocatalysts over long-term electrochemical reactions plays an important role in their overall electrochemical performance. The stabilities of both 2Co@NF and 2NMC@NF were assessed through long-term CA measurements (24 h) as shown in Figure 4f. As shown in the current–time (*I*–*t*) plot in Figure 4f, it is evident that current density decreased significantly with time, reaching 11.7 mA cm^–2^ after 24 h CA at 1.5 V vs RHE for 2NMC@NF, equal to a 67% drop in the current density. This current decay is typical for organic oxidation in batch cells, where benzyl alcohol is progressively consumed and converted to oxidized products and intermediates, leading to a continuous decrease in current over time.^64^ 2Co@NF displayed a 90% current density drop after 24 h of electrolysis at 1.5 V vs RHE, which indicated that most of the benzyl alcohol had been converted. From the LSV polarization plots after 24 h of CA at 1.5 V vs RHE in the spent electrolyte, 2NMC@NF showed the requirement of an additional 229 ± 5 mV potential to achieve a current density of 10 mA cm^–2^ compared to its as-synthesized state (Figure S8). In contrast, 2Co@NF required an additional 242 ± 15 mV under the same conditions. It is important to establish that the pronounced decrease in activity was due to electrocatalyst deactivation or the conversion of benzyl alcohol during the prolonged 24 h CA stability test. Hence, the spent electrocatalysts were tested in a fresh electrolyte after washing and drying under ambient conditions to remove the physically adsorbed intermediates and products. For 2NMC@NF, there was only a marginal 2 ± 1 mV increase of potential to attain 10 mA cm^–2^ current density compared to the as-synthesized electrocatalyst, whereas 2Co@NF required a higher overpotential increase of 39 ± 3 mV to reach the same current density (Figure 4f inset, dashed plots, red is 2Co@NF, blue is 2NMC@NF). These results suggest that 2NMC@NF retained its catalytic stability better than 2Co@NF after prolonged operation, and the increase of overpotential in the used electrolyte is not due to the electrocatalyst degradation but due to the conversion of the benzyl alcohol to the products. To elucidate the individual roles of Ni and Mn toward stability, the LSV performances of 2MC@NF and 2NC@NF were evaluated after 24 h of CA stability at 1.5 V vs RHE in new electrolytes. Figure S9a,b shows that the performance of 2NC@NF was enhanced compared to 2MC@NF, but its stability was inferior. The activity of poststability 2MC@NF slightly improved at higher current density which could be due to possible *in-situ* reconstruction that improves access to Co active sites.

The long-term BAOR stability was further probed by subjecting all electrocatalysts to 1000 CV cycles between 0.6 and 1.8 V vs RHE at 50 mV s^–1^ in 1 M KOH + 0.1 M benzyl alcohol while continuously recirculating electrolyte with a peristaltic pump to mitigate surface poisoning by oxidation products. Under these conditions, the Co^3+/^Co^2+^ reduction peak area, which reflects the density of regenerable Co^3+^ active sites, was integrated after every 100 cycles to track Co active site deactivation (Figure S10a). For 2Co@NF, the reduction peak area decreased by 23% from 30.1 to 23.2 mC cm^–2^ after 1000 cycles, indicative of active-site loss or surface area loss via agglomeration. Among the electrocatalysts, 2MC@NF exhibited the smallest loss in Co^3+/^Co^2+^ charge (1.2 mC·cm^–2^ loss (∼7% decrease) after 1000 cycles) and, despite its lower initial activity, it maintained nearly constant performance over cycling (Figure S10b). 2NC@NF showed improved stability relative to 2Co@NF but remained less stable than 2NMC@NF and 2MC@NF, underscoring the stabilizing influence of Mn incorporation into the CoOOH framework.

In electrocatalysis, the versatility of electrocatalysts, especially for different substrates is highly desirable. CoOOH has been extensively used for different anodic reactions such as the OER, BAOR and even glycerol electrooxidation.^10,22^ With this in mind, we evaluated 2NMC@NF for the electrooxidation of well-explored alcohols, such as ethanol (EOR) and ethylene glycol (EGOR), in addition to benzyl alcohol oxidation (BAOR). Since aliphatic alcohols such as ethanol (EtOH) and ethylene glycol (EG) are highly soluble in 1 M KOH, we probed 2NMC@NF performance at higher alcohol concentration (1 M KOH containing 0.2 M of the respective alcohol). For a fair comparison, BAOR was examined in 1 M KOH and 0.2 M benzyl alcohol under identical conditions. First, the unchanged BAOR activity of 2NMC@NF upon increasing benzyl alcohol to 0.2 M indicates saturation of the catalytically active sites at 0.1 M benzyl alcohol. Relative to BAOR, the onset potentials for EOR and EGOR are shifted positively by a similar 16 mV, while the potentials required to reach 10 mA cm^–2^ are 65 and 79 mV higher, respectively (Figure S11a). At a higher current density of 50 mA cm^–2^, the potential gaps with respect to BAOR increase to 115 mV for both EOR and EGOR, underscoring the intrinsically faster BAOR kinetics on 2NMC@NF. When benchmarked against the oxygen evolution reaction (OER) in 1 M KOH only, the alcohol oxidations display substantial potential requirements. The onset potential of the OER is 202 mV more positive than that for BAOR, and an additional 254 mV is required to reach 10 mA cm^–2^, highlighting the advantage of replacing OER with organic electrooxidation on this electrocatalyst. Collectively, these results demonstrate that 2NMC@NF is not only highly active for BAOR but also competent for other organic anodic reactions such as EOR and EGOR.

Additionally, the 12 h stability of 2NMC@NF (Figure S11b) in 1 M KOH with different alcohols was investigated. Under a constant potential of 1.5 V vs RHE in 1 M KOH + 0.2 M alcohol, all three alcohols exhibit a gradual decay in current density over 12 h, consistent with the progressive consumption of the substrate and accumulation of oxidized products rather than rapid electrocatalyst deactivation. In contrast, in electrolyte containing only 1 M KOH, 2NMC@NF displays excellent stability for OER at a slightly higher constant potential of 1.6 V vs RHE over 12 h, indicating that 2NMC@NF. The Tafel slopes (Figure S11c) extracted from the corresponding polarization curves in Figure S11a align with these observations, revealing lower slopes (faster apparent charge-transfer kinetics) for BAOR, EOR, and EGOR than for the OER

### Temperature-Dependent and Diffusion-Controlled Kinetics Study of 2Co@NF and 2NMC@NF during BAOR

For temperature-dependent analysis, LSV polarization curves in Figure 5a,b for 2NMC@NF and 2Co@NF, showed progressively increased activity with temperature (from 303 to 353 K), respectively, demonstrating that BAOR is thermally promoted, likely due to enhanced mass-transport and reaction kinetics at elevated temperatures.

**Figure 5:**
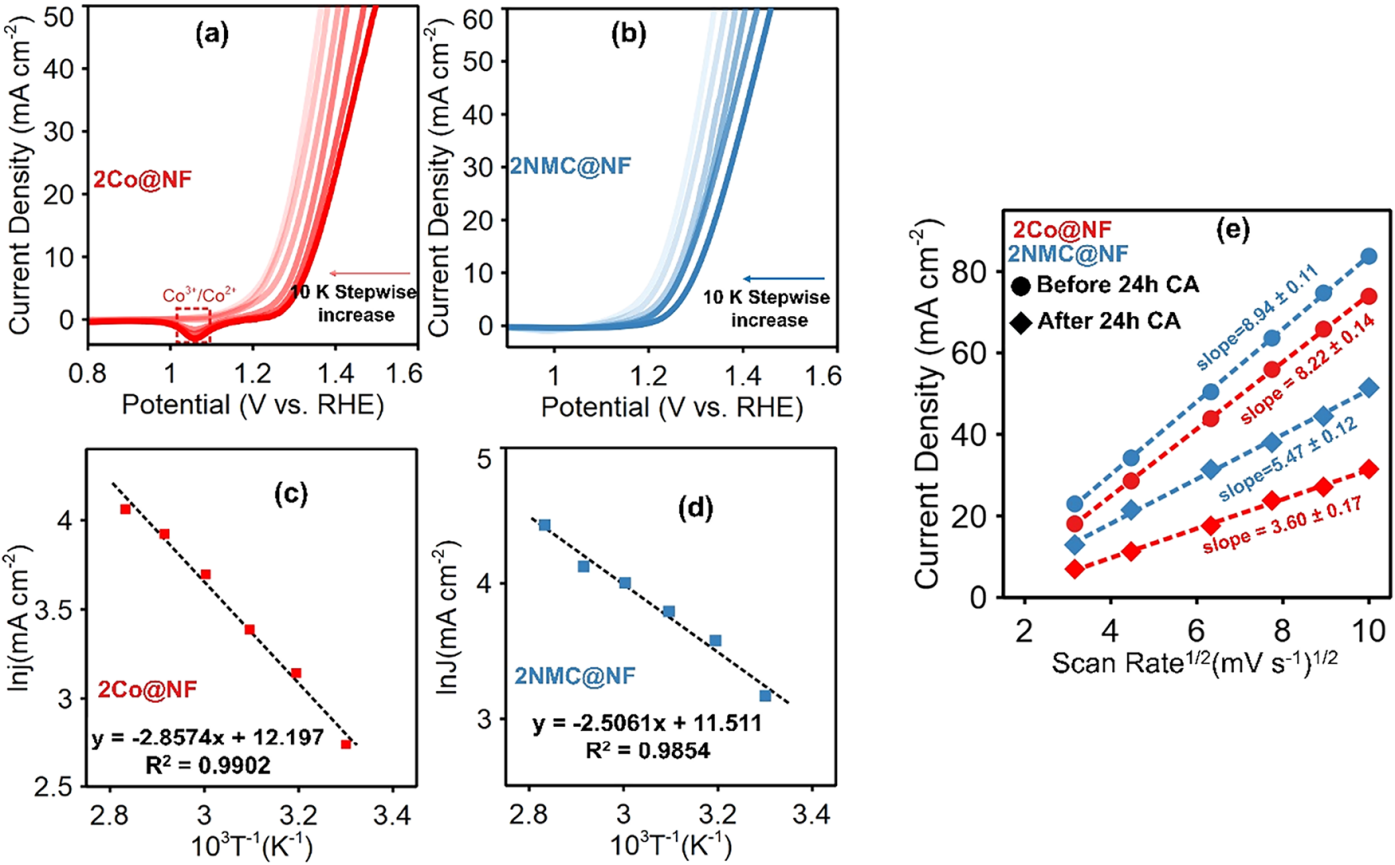
LSV polarization plots at a different temperature of (a) 2Co@NF, (b) 2NMC@NF, Arrhenius plots at temperatures of 30 to 80 °C with constant 700 rpm stirring of (c) 2Co@NF and (d) 2NMC@NF, and (e) Randles–Sevcik plot of 2Co@NF and 2NMC@NF before and after 24 h CA at a constant potential of 1.5 V vs RHE. For the Randles–Sevcik fits in (e), the regression equations are 2NMC@NF before CA, *y* = 8.941*x* – 5.611; 2NMC@NF after CA, *y* = 5.465*x* – 3.732; 2Co@NF before CA, *y* = 8.224*x* – 8.007; and 2Co@NF after CA, *y* = 3.598*x* – 4.672. Slopes in the Randles–Sevcik plots are reported as value ± error, where the error represents the standard error of the slope obtained from linear regression. (All measurements in 1 M KOH + 0.1 M benzyl alcohol).

However, for each temperature measured, the activity of 2NMC@NF was found to be higher than that of 2Co@NF at the reference potential of 1.38 V vs RHE, indicating the enhanced electrocatalytic performance of 2NMC@NF even above ambient temperatures. The Arrhenius plot for both 2Co@NF and 2NMC@NF electrocatalysts (Figure 5c,d) from LSV plots in Figure 5a,b (details in Experimental Section and Supporting Information (SI)) indicates a linear relationship between ln *J* and 1/*T*, suggesting that the reaction mechanism remains unchanged at higher temperatures. The calculated *E*_a_ values for 2Co@NF and 2NMC@NF were 23.76 and 20.83 kJ mol^–1^, respectively. The 2NMC@NF required a lower activation energy than 2Co@NF for the electrocatalytic BAOR, manifesting enhanced kinetics of electrocatalytic oxidation of benzyl alcohol for the former electrocatalyst, resulting from the synergistic effect of Co^3+^ and Ni^3+^ in 2NMC@NF.

The electrocatalytic kinetics of both electrocatalysts were further evaluated by analyzing the dependence of peak current density (*I*_p_) on the scan rate (*v*). According to Randles–Sevcik postulation, the effect *v* on *I*_p_ for redox reactions can give insight into the reaction kinetics and diffusion coefficient of electrocatalysts.^64^ To this end, CV measurements were performed at a potential range of 0.7 to 1.6 V vs RHE and the scan rate was varied from 10 mV s^–1^ to 100 mV s^–1^ (Figure S12). As the scan rate increased, the peak current density increased correspondingly due to higher concentration gradients at the electrocatalyst interface that enhanced mass transport and increased the flux of redox-active species to the surface. In addition, the anodic oxidation peaks shifted to more positive potentials and the cathodic reduction peaks to more negative potentials with increasing scan rate, characteristic of a quasi-reversible charge–discharge process for both electrocatalysts.^65,66^ Figure 5e shows Randles–Sevcik plots of peak current versus *v* ^1/2^ before and after 24 h CA for 2Co@NF and 2NMC@NF electrocatalysts, where the linear dependence of current density on the square root of scan rate indicates diffusion-controlled behavior.^67^ The slopes of the linearized plots quantify the combined effects of electrochemically active surface area, charge-transfer kinetics, and diffusion of electroactive species, with larger slopes corresponding to more efficient mass transport and faster electron transfer.^64^ The 2NMC@NF electrocatalyst exhibited the steepest slope (8.94 ± 0.11 mA·cm^–2^ (mV·s^–1^)^−1/2^ (*R* ^2^ = 0.9993)), indicating a higher diffusion coefficient and faster electron transfer kinetics. In contrast, 2Co@NF showed a lower slope (8.22 ± 0.14 mA·cm^–2^ (mV·s^–1^)^−1/2^ (*R* ^2^ = 0.9991)), reflecting slower charge transfer and a lower diffusion coefficient. After 24 h of continuous CA at 1.5 V vs RHE, the slope of 2Co@NF dropped to 3.60 ± 0.17 mA·cm^–2^ (mV·s^–1^)^−1/2^ (*R* ^2^ = 0.994), a 56% decrease, indicating severely hindered charge transfer. In comparison, 2NMC@NF showed a smaller decrease from 8.9407 to 5.47 ± 0.12 mA·cm^–2^ (mV·s^–1^)^−1/2^ (0.991), a 39% decrease, suggesting better retention of charge-transfer efficiency and diffusion capability over prolonged operation. This stability demonstrated that the transport of electroactive species to the electrocatalyst surface was less hindered by adsorbed oxidation products, allowing 2NMC@NF to retain its catalytic performance more effectively over time.

### Post-CA Physical Characterization of 2Co@NF and 2NMC@NF

FESEM, HAADF-STEM, PXRD, XPS, and ICP-OES techniques were employed to evaluate any possible electrocatalyst structural and/or electronic changes or reconstruction after 24 h of CA BAOR. FESEM analysis confirmed that the 2D morphology was retained after the CA test for both 2Co@NF and 2NMC@NF (Figure 6a,6b). However, with closer observation, there is an agglomeration of the microflowers of 2Co@NF after 24 h of CA (Figure 6a). This agglomeration reduced the surface area and thereby made the nanosheets less accessible, leading to reduced activity observed after 24 h of CA BAOR. The EDS quantification in Figure S13a showed the atomic ratio of Ni/Mn/Co in 2NMC@NF to be 0.31:0.39:1 after 24 h of CA, indicating a 68% decrease in Mn content relative to Ni in the as-deposited electrocatalyst. HAADF-STEM imaging and STEM-EDS mapping (Figure 6c) indicated that Co, Ni, and Mn are all present on the same nanosheet, but their distributions are not equally uniform after 24 h of CA. Ni is more diffuse, whereas Mn is more localized and concentrated toward the central portion of the nanosheet, indicating structural transformation. This spatial inhomogeneity is consistent with preferential Mn leaching from exposed surface regions during 24 h of BAOR, leading to a Mn-enriched core and Mn-depleted edges, corroborated by the reduced intensity in EDS and STEM-EDX quantifications in Figure S13a,b. Post-CA bulk characterization by PXRD showed that the overall bulk structures of 2Co@NF and 2NMC@NF remained largely unchanged (Figure 6d). Additionally, there were no noticeable changes in *ex-situ* Raman characterizations of spent 2Co@NF and 2NMC@NF (Figure S14), indicating that this transformation is primarily compositional and morphological rearrangements.

**Figure 6:**
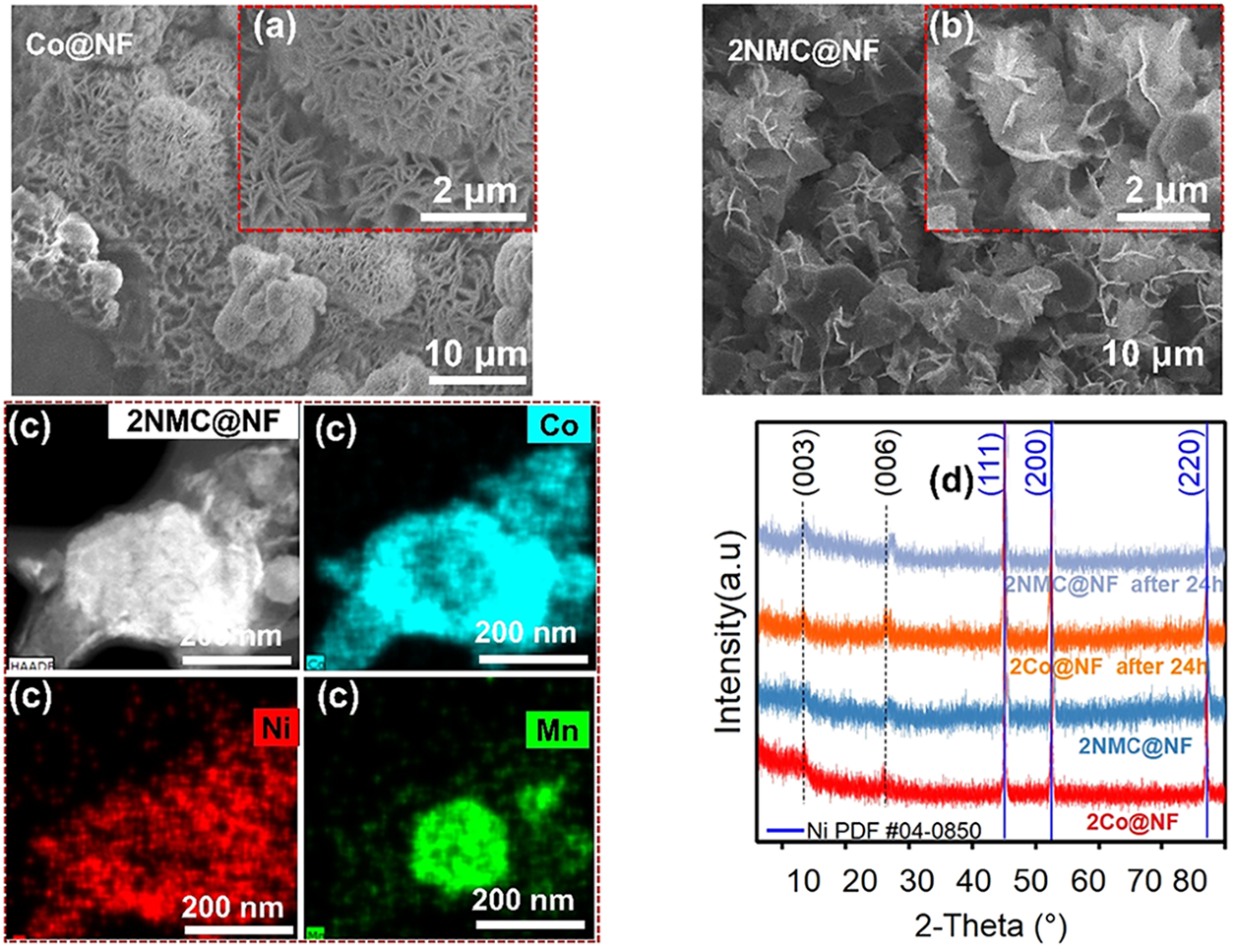
FESEM images after 24 h CA at a constant potential of 1.5 V vs RHE in 1 M KOH + 0.1 M benzyl alcohol of (a) 2Co@NF, (b) 2NMC@NF, (c) HAADF-STEM mapping after 24 h CA at constant potential of 1.5 V vs RHE of 2NMC@NF, and (d) PXRD patterns of 2Co@NF, 2NMC@NF before and after 24 h CA at a constant potential of 1.5 V in 1 M KOH + 0.1 M benzyl alcohol.

To possibly identify changes in oxidation states of Co, Mn, and Ni, 2Co@NF, 2NC@NF, 2MC@NF, and 2NMC@NF electrocatalysts after 24 h CA at 1.5 V vs RHE were characterized by XPS. The Ni 2p peaks of the 2NC@NF and 2NMC@NF after 24 h CA maintained the same peak shapes as the pristine electrocatalysts but shifted positively (+0.4 and 0.2 eV, respectively), suggesting a slight increase in the overall Ni oxidation state (Figure 7a). *Ex-situ* XPS characterization ruled out the presence of Ni^4+^, as this oxidation state is thermodynamically unstable at open-circuit potential and will readily reduce to NiOOH (Ni^3+^) or even Ni­(OH)_2_ (Ni^2+^) upon removal of the applied bias.^68^ Therefore, the slight increase in average binding energy is more plausibly attributed to the enhanced generation of Ni^3+^ species during the prolonged stability test. The high-resolution Co 2p XPS spectra of 2Co@NF, 2MC@NF, 2NC@NF, and 2NMC@NF (Figure 7b) can be fitted with 4 peaks similar to the pristine electrocatalysts, indicating that the Co active sites remained dominant after electrocatalytic oxidation. The peak-to-peak area ratio of 780.5 to 779.5 eV peaks in 2Co@NF showed an observable decrease after 24 h CA, indicating possible surface reconstruction or transformation. No significant shifts in binding energies were observed in Figure 7b.

**Figure 7:**
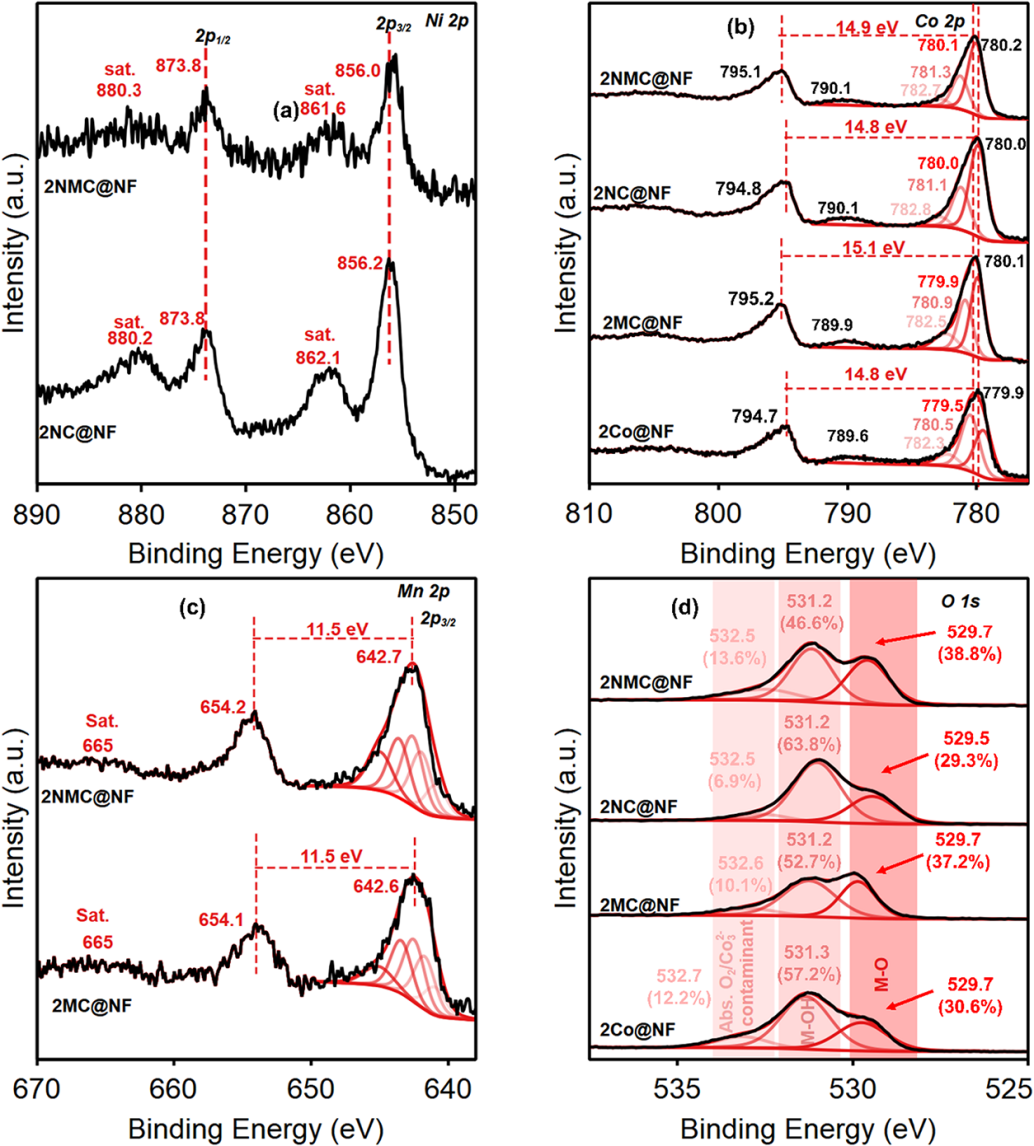
XPS after 24 h CA at constant potential of 1.5 V vs RHE of (a) Ni 2p for as-synthesized 2NC@NF and 2NMC@NF, (b) Co 2p for as-synthesized 2Co@NF, 2MC@NF, 2NC@NF, and 2NMC@NF, and (c) Mn 2p for as-synthesized 2MC@NF and 2NMC@NF, (d) O 1s for as-synthesized 2Co@NF, 2MC@NF, 2NC@NF, and 2NMC@NF. (Electrolyte: 1 M KOH + 0.1 M benzyl alcohol).

The Mn 2p spectra of 2MC@NF and 2NMC@NF after 24 h of CA at a constant potential of 1.5 V vs RHE were peak-fitted using the same fitting model and parameters as pristine 2MC@NF and 2NMC@NF, suggesting that the chemical and electronic properties of Mn remained stable throughout the prolonged electrolysis (Figure 7c). The O 1s spectra of 2MC@NF, 2NC@NF, and 2NMC@NF after 24 h of CA (Figure 7d) closely matched pristine electrocatalysts. However, the intensity of the M−O relative to M−OH components increased after 24 h CA for 2MC@NF and 2Co@NF, consistent with further conversion of residual Co­(OH)_2_ to CoOOH during the stability test but remained essentially unchanged for 2NMC@NF and decreased for 2NC@NF. Provided with the small positive shift in the overall Co 2p_3/2_ binding energy (779.7 to 779.9 eV) in 2Co@NF, *in-situ* oxidation during stability testing was limited.

XPS quantification was used to determine the surface compositions of Ni, Mn, and Co for new and post-CA electrocatalysts (Table S3). For 2NMC@NF, the Ni/Mn atomic ratio showed a reduction relative to Co after 24 h of CA. For detailed interpretation of possible metal leaching post-BAOR, the %weight of all metals at the surface and bulk of the electrocatalysts by XPS and ICP-OES was compared. Figure 8a presents XPS-derived weight percentages of Ni, Mn, and Co for pristine and post-CA (spent) electrocatalysts, revealing pronounced Mn depletion in both 2MC@NF (44.11 to 20.94 wt %) and 2NMC@NF (28.19 to 15.93 wt %). Complementary ICP-OES-derived normalized weight fractions (Figure 8b) for the chemical composition of electrocatalysts after CA show a consistent decrease in bulk Mn content relative to Co and Ni, confirming Mn loss from both 2MC@NF and 2NMC@NF. In 2MC@NF, Mn wt% decreased from 30.93 to 19.07 after 24 h CA, whereas 2NMC@NF exhibited a 4.73 wt % decline, further supporting selective Mn leaching during prolonged operation. However, ICP-OES measurements of the corresponding spent electrolyte (Table S4) showed no detectable Mn, indicating that leached Mn did not remain in solution. To investigate potential Mn redeposition, all CFPs used as counter electrodes during CA testing were physically examined. Figure S15 revealed a distinct surface layer on CFPs paired with 2NMC@NF and 2MC@NF, absent in those with 2Co@NF or 2NC@NF, confirming selective Mn species redeposition from Mn-containing electrocatalysts. *Ex-situ* Raman measurement of the counter electrode used for 2NMC@NF 24 h CA stability confirmed the presence of Mn_3_O_4_ ^69^ deposited on CFP, validating Mn leaching and redeposition on the counter electrode during prolonged test (Figure S16). Collectively, these post-CA characterization and activity trends indicate that initial Mn incorporation generates a surface MnO_2_ layer that partially blocks Co active sites yet enhances CoOOH stability, while subsequent Mn leaching during BAOR re-exposes Co sites and contributes to the improved post-CA activity of 2MC@NF.

**Figure 8:**
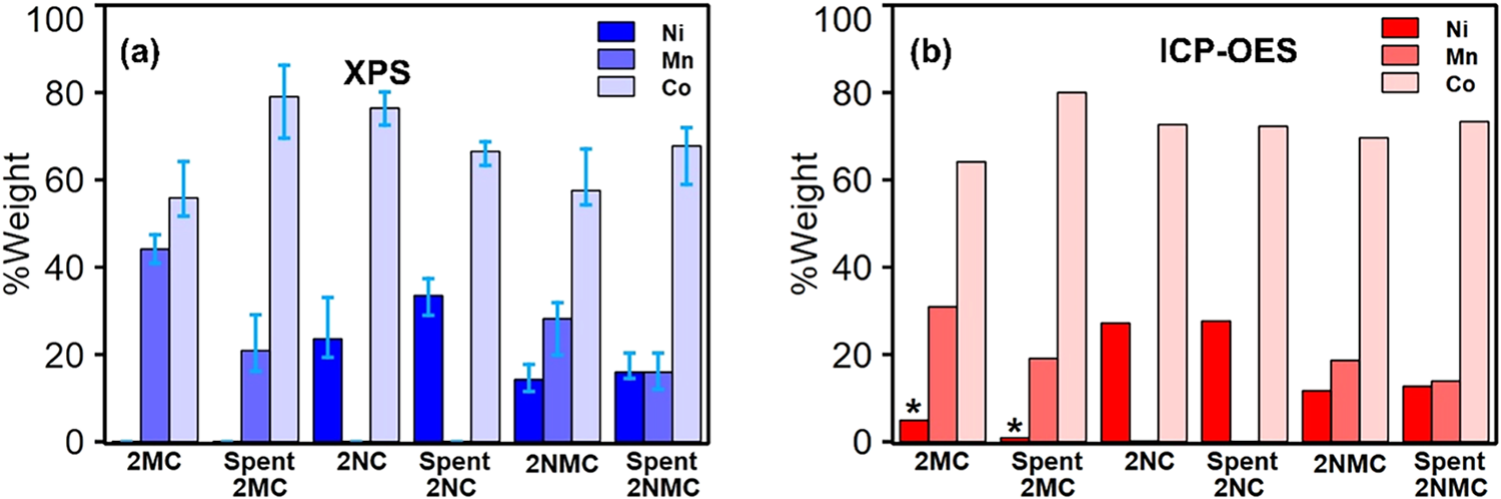
Estimation of weight percentages of Ni, Mn, and Co metals before and after 24 h CA at 1.5 V vs RHE (1 M KOH + 0.1 M benzyl alcohol, 1.5 V vs RHE) from (a) XPS characterization and (b) ICP-OES. *Residual Ni from NF.

### Liquid Product Analysis of Benzyl Alcohol Electrooxidation by 2Co@NF and 2NMC@NF

24 h CA experiments were performed to evaluate the BAOR performance of 2Co@NF and 2NMC@NF by HPLC. Detailed HPLC conversion plot from 24 h CA experiment is shown in See Figure S17.

As shown in Figure 9a, over time, the concentration plots for both 2Co@NF and 2NMC@NF exhibited a decrease in benzyl alcohol and an increase in the concentration of benzoic acid, consistent with benzyl alcohol electrooxidation. For 2NMC@NF, the benzyl alcohol concentration decreases approximately linearly over the first 8 h of electrolysis, reaching more than 70% conversion. Beyond 8 h, the conversion rate slows and approaches 92.9% after 24 h of CA, consistent with mass-transport limitations at low substrate concentration. At 8 h, the benzyl alcohol concentration is about 27.2 mM, and this diminished concentration is responsible for the reduced conversion rate in the later stages of the reaction. In comparison, 2Co@NF shows a similar conversion behavior during the first 4 h, but its conversion rate declines more after 8 h, likely due to electrocatalyst deactivation or the loss of active sites. The selectivity of benzoic acid was consistently greater than 80% at every interval of the CA measurement, reaching approximately 93% after 15h CA, indicating low partial conversion of benzyl alcohol to benzaldehyde (Figure 9b). This underscores the effectiveness of 2NMC@NF for the full oxidation of benzyl alcohol to benzoic acid. Generally, the electrooxidation pathway of benzyl alcohol follows that benzyl alcohol first oxidizes to benzaldehyde, and then benzaldehyde is subsequently oxidized to benzoic acid. However, from the HPLC analysis, a low concentration of benzaldehyde was constantly observed to be formed. For both 2NMC@NF and 2Co@NF, benzaldehyde is formed predominantly at initial hours, accounting for 15.6% and 10.1% of the total products after 4 and 8 h of CA in 2NMC@NF (Figure 9c, yields normalized to 100%). For 2NMC@NF, the benzaldehyde fraction then dropped to approximately 3.9% and 5.3% at 15 and 24 h, consistent with further oxidation to benzoic acid at longer electrolysis times. The overall Faradaic efficiency for benzoic acid formation on 2NMC@NF reached 91.4% after 24 h of CA (Figure 9d).

**Figure 9:**
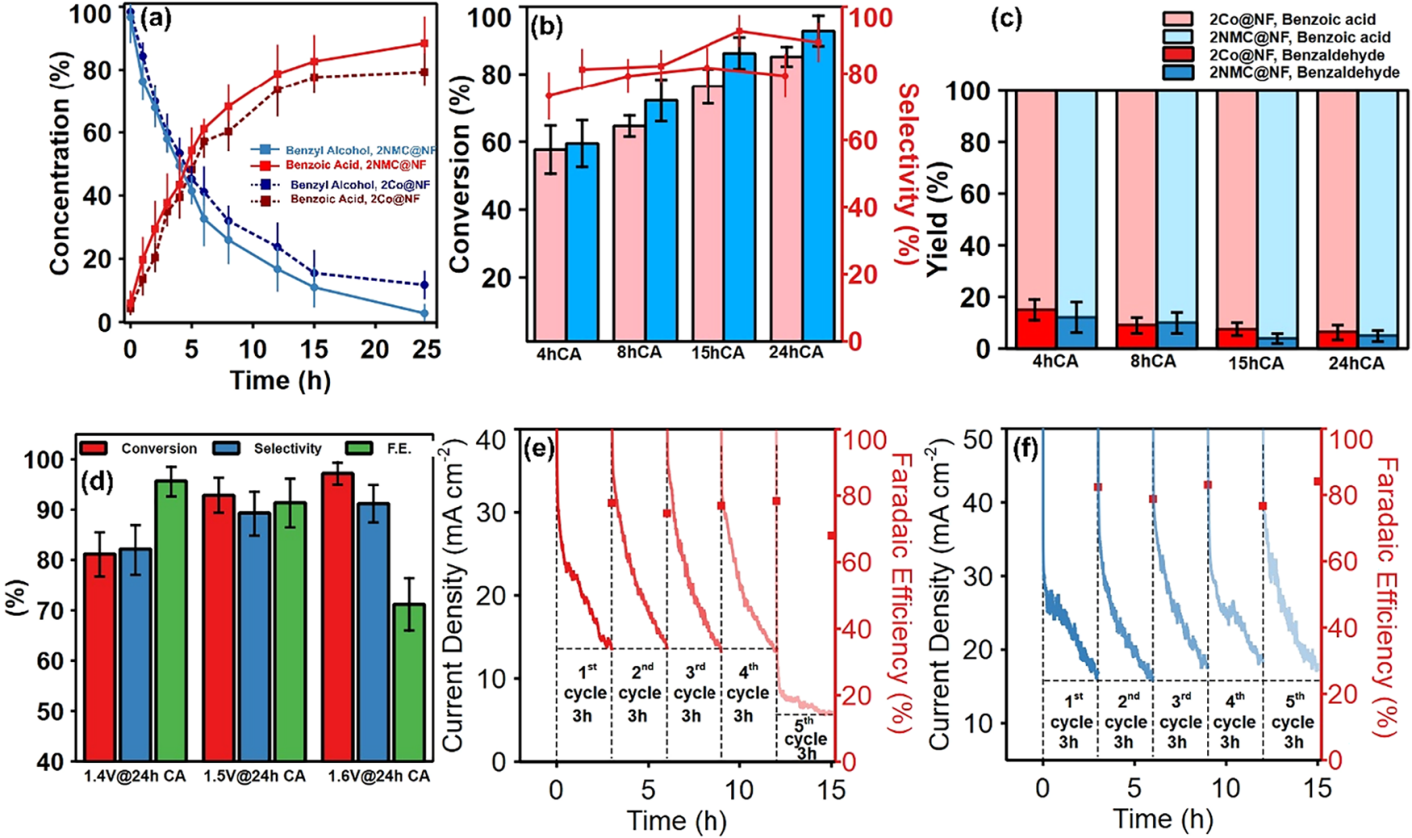
(a) Concentration profile of benzyl alcohol and benzoic acid by 2Co@NF and 2NMC@NF over 24 h CA at 1.5 V vs RHE. (b) Benzyl alcohol conversion plots of 2Co@NF (red) and 2NMC@NF (blue), and selectivity of 2Co@NF (circle) and 2NMF@NF (square) at selected CA time. (Error bars from three independent experiments), (c) Normalized % yield plots of 2Co@NF and 2NMC@NF for benzoic acid and benzaldehyde at selected CA time. (Error bars from three independent experiments), (d) benzyl alcohol conversion, benzoic acid selectivity, and benzoic acid Faradaic efficiencies during BAOR by 2NMC@NF at 1.4, 1.5., and 1.6 V CA. All measurements were done in 1 M KOH + 0.1 M Benzyl alcohol, (e) BAOR recyclability test using 2Co@NF at constant potential of 1.5 V vs RHE, 3 h CA per cycle. (f) BAOR recyclability test using 2NMC@NF at constant potential of 1.5 V vs RHE, 3 h CA per cycle. (Error bar from 3 independent readings).

The potential is an important parameter in electrochemical reactions, as it can affect the electrochemical performance of the electrocatalysts. Therefore, the influence of potential on BAOR for 2NMC@NF electrocatalyst was explored by performing CA for benzyl alcohol conversion at 1.4 and 1.6 V in addition to 1.5 V vs RHE. At 1.6 V, benzyl alcohol conversion reached 97.2% after 24 h, but the FE for benzoic acid formation dropped by about 13% relative to that at 1.5 V vs RHE, likely due to the onset of competing OER at this higher potential (Figure 9d). In contrast, at 1.4 V, the FE was highest because the OER was negligible, but the overall conversion remained low.

Additionally, to probe activity and stability differences between pristine 2Co@NF and 2NMC@NF, recyclability tests (Figure 9e,f) were performed by conducting five consecutive 3 h CA runs at 1.5 V vs RHE, reusing the same electrodes with fresh electrolyte (1 M KOH + 0.1 M benzyl alcohol) for each BAOR cycle. 2Co@NF showed sustained performance in the first 4 cycles; however, a noticeable reduction in the current density was observed during the fifth cycle, translating into a gradual loss of BAOR activity. Because the electrolyte was replaced between cycles, accumulation of products or byproducts at the electrode–electrolyte interface can be largely excluded as the origin of this performance decay, suggesting the onset of intrinsic catalyst deactivation. 2NMC@NF demonstrated excellent stability over 5 cycles with sustained current density and Faradaic efficiency above 80%.

Apart from the effect of applied potential, extrinsic parameters, such as reactant concentrations and operating conditions, critically influence BAOR activity. Elevated temperature not only enhances the kinetics of BAOR (as highlighted in Figure 5a–d) but can alter major product selectivity, while varying benzyl alcohol concentrations affects reaction rates and subsequently the product. These factors are critical to evaluate and subsequently optimize BAOR performance. Using 2NMC@NF, we performed 24 h CA at a constant potential of 1.5 V vs RHE in 1 M KOH and different concentrations of benzyl alcohol (0.05 and 0.2 M benzyl alcohol) and at different temperatures (30 and 40 °C) (Figure S18a–d). At lower benzyl alcohol concentration (0.05 M) (Figure S18a), benzyl alcohol conversion proceeds at a much faster rate, reaching 97% after 24 h CA. Additionally, selectivity toward benzoic acid continues to increase, reaching 90% after 6 h CA. From a catalytic point of view, this trend is plausible because after benzyl alcohol depletion, more reaction time is focused on subsequent conversion of benzaldehyde. At higher benzyl alcohol concentration (0.2 M) (Figure S18b), while FE remained barely affected, however, conversion and selectivity reduced (73.6 and 85.8% after 24 h CA, respectively). Increasing the temperature favored increased selectivity, but FE tends to slightly reduce at higher temperature due to increased charge applied due to increased charge applied-to-conversion ratio at longer CA time (Figure S18c,d). However, after 24 h CA at 40 °C, 99% of benzyl alcohol was converted and selectivity reached approximately 96%. Collectively, these results demonstrate superb BAOR performance of 2NMC@NF comparable to that of the state-of-the-art for Co-based electrocatalysts (Table S5).

## Conclusions

In summary, a model CoOOH-based electrocatalyst was synthesized to elucidate how transition-metal impurities impact activity and stability during BAOR. Incorporation of Ni and Mn into CoOOH via a facile electrodeposition–CV activation protocol yielded a trimetallic 2NMC@NF electrocatalyst, which exhibits enhanced BAOR performance relative to 2Co@NF due to tunable electronic and structural properties. Particularly, Ni incorporation tuned the CoOOH electronic structure and introduces additional Ni^3+^ redox sites that accelerate BAOR kinetics, whereas Mn plays a primarily passivating role: surface MnO_2_ slightly suppresses initial activity by blocking Co sites but mitigates Co dissolution and active-site loss under prolonged chronoamperometry. Poststability electrochemical measurements, together with bulk and surface characterization, revealed partial Mn leaching and redeposition as Mn_3_O_4_ while largely preserving the CoOOH framework. As a result, 2NMC@NF delivers high and durable benzyl alcohol oxidation to benzoic acid, achieving 92.9% benzyl alcohol conversion and 89.3% selectivity under ambient conditions. These findings provide insights into the individual and cooperative effects of trace Ni and Mn, representative of common handling or electrolyte-borne contaminants, on the structure–activity relationships of CoOOH-based electrocatalysts toward electrooxidation of alcohols like benzyl alcohol.

## Supplementary Material



## Notes

CV oxidation plot, FESEM images of as-synthesized hydroxides; SEM-EDS and STEM-EDX plots of CoOOH and Ni and/or Mn in CoOOH; Raman characterizations of hydroxides and oxyhydroxides; XPS characterization of hydroxides; ECSA determination; LSV curves of the control samples; current density vs potential at different scan rates for Randles–Sevcik plots, CV cycling plots; XPS and ICP-OES quantification of spent electrodes; HPLC chromatograph plot; and BAOR conversion plots at different temperatures and alcohol concentrations (PDF)
