# Supplementary material for: Elucidating the Structural and Electronic Effects of Ni and Mn Cationic Incorporation on CoOOH for Efficient Benzyl Alcohol Electrooxidation
Source: ACS Appl Energy Mater. 2026 Apr 6;9(8):4814–29. doi: 10.1021/acsaem.5c04095 (PMC13126441; doi:10.1021/acsaem.5c04095)
Supplement: Supplementary file 1 [file ae5c04095_si_001.pdf]

Supporting Information

Elucidating the Structural and Electronic Effects of Ni and Mn Cationic Incorporation on CoOOH for Efficient Benzyl Alcohol Electrooxidation

Emmanuel Aransiola[a], Sahanaz Parvin[a], Mohamed Ammar[a], Rachel Smith[a], Lihua Zhang[b], Nishu Devi[c], Barbara R. Evans[c], Juliane Weber[c], and Jonas Baltrusaitis[a*]

[a]*Department of Chemical and Biomolecular Engineering, Lehigh University, 111 Research Dr., Bethlehem, PA 18015, USA*
[b]*Brookhaven National Laboratory, Center for Functional Nanomaterials, Bldg. 735, Upton, New York 11973-5000, United States, USA*
[c]*Oak Ridge National Laboratory, Chemical Sciences Division, Oak Ridge, TN 37831, USA*

**Corresponding author* job314@lehigh.edu, *Phone: +1-610-758-6836*

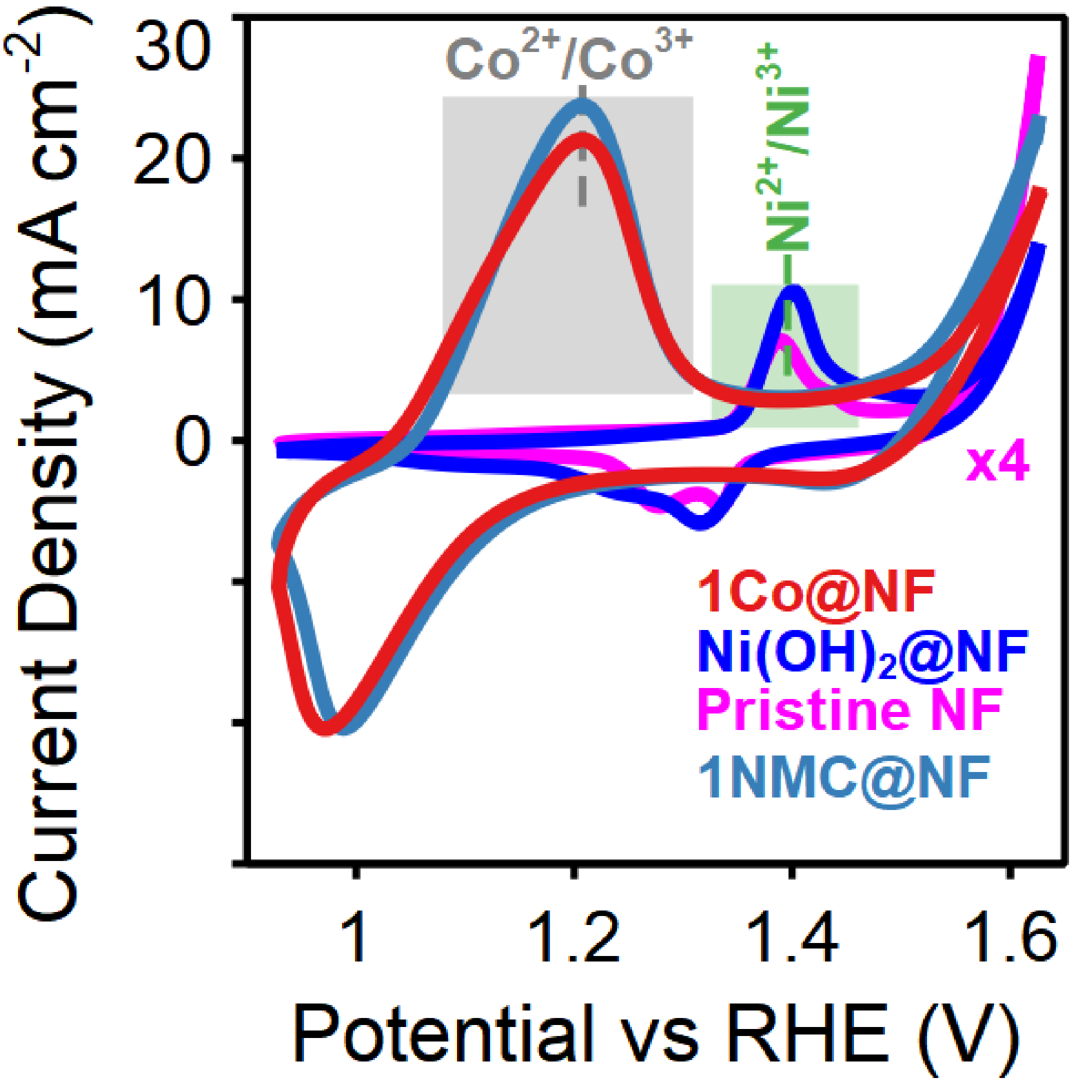

Figure S1. CV plots in 1 M KOH for as-deposited 1NMC@NF, 1Co@NF (flattened NF), $Ni(OH)_2$@NF, and Pristine NF. No Ni^{2+}/Ni^{3+} peak is observed in 1NMC@NF and 1Co@NF, suggesting no catalytic effects due to the NF Substrate used.
Reference Electrode: Hg/HgO/OH-; Counter Electrode: Carbon fiber paper.

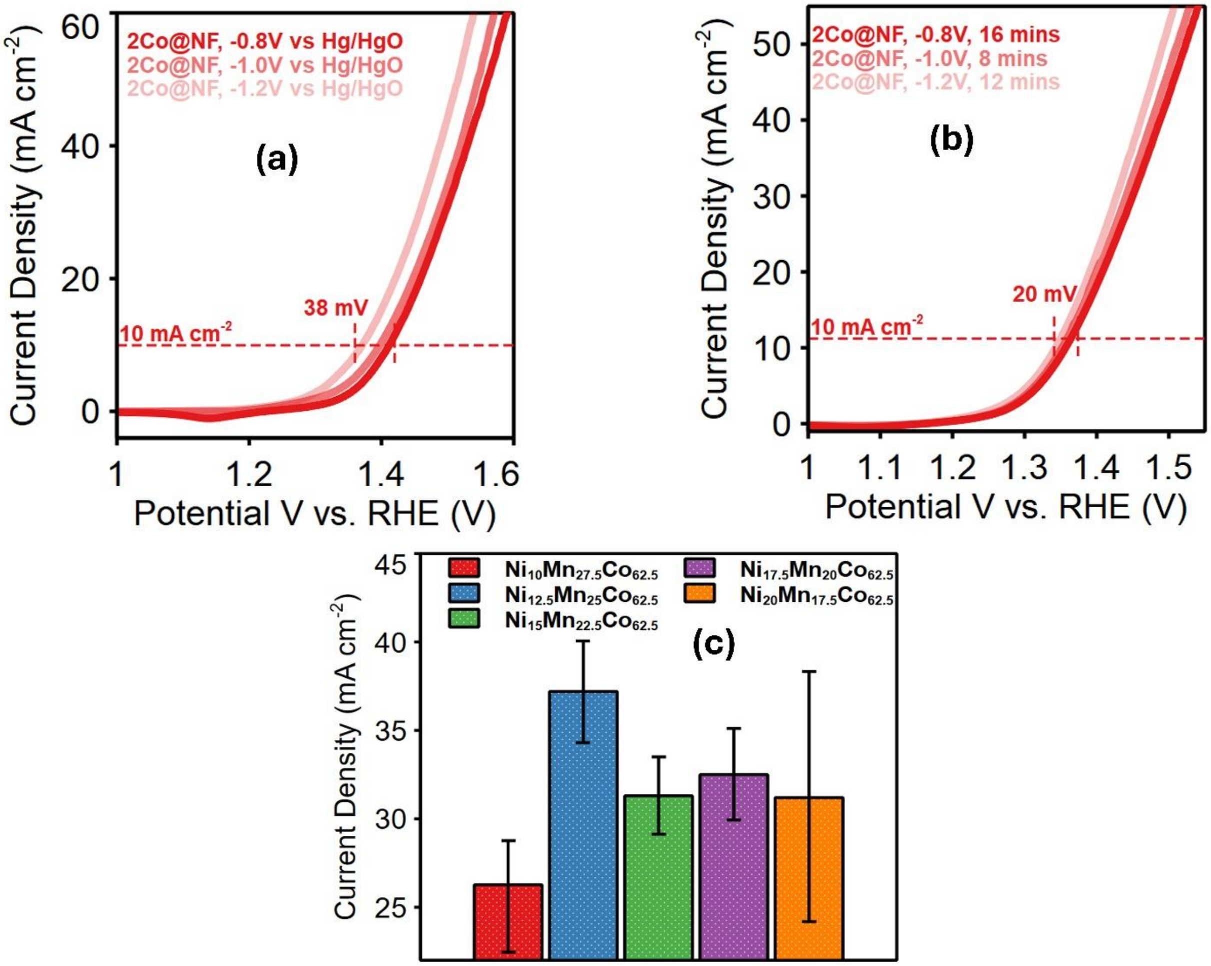

Figure S2: linear polarization plots of 2Co@NF electrocatalyst electrodeposition optimization (a) at different potentials and (b) at different electrodeposition times in the presence of 0.1 M BzA. Optimization of precursor incorporation (c) measured current density of 2NMC@NF for different Ni and Mn precursor concentrations in solution obtained at 1.4 V vs RHE. (Error bars were calculated from 3 different LSV measurements). The concentration of Co precursor was maintained at 62.5 mM.

Table S1: Electrocatalyst Nomenclature

**Electrodes**	**Nomenclature**	**CV Oxidation/Conversion in 1M KOH**
1Co@NF	Cobalt hydroxide	No
1NMC@NF	Nickel and manganese incorporated cobalt hydroxide	No
2Co@NF	cobalt oxyhydroxide	Yes
2NMC@NF	Nickel and manganese incorporated cobalt oxyhydroxide	Yes
2NC@NF	Nickel incorporated cobalt oxyhydroxide	Yes
2MC@NF	Manganese incorporated cobalt oxyhydroxide	Yes

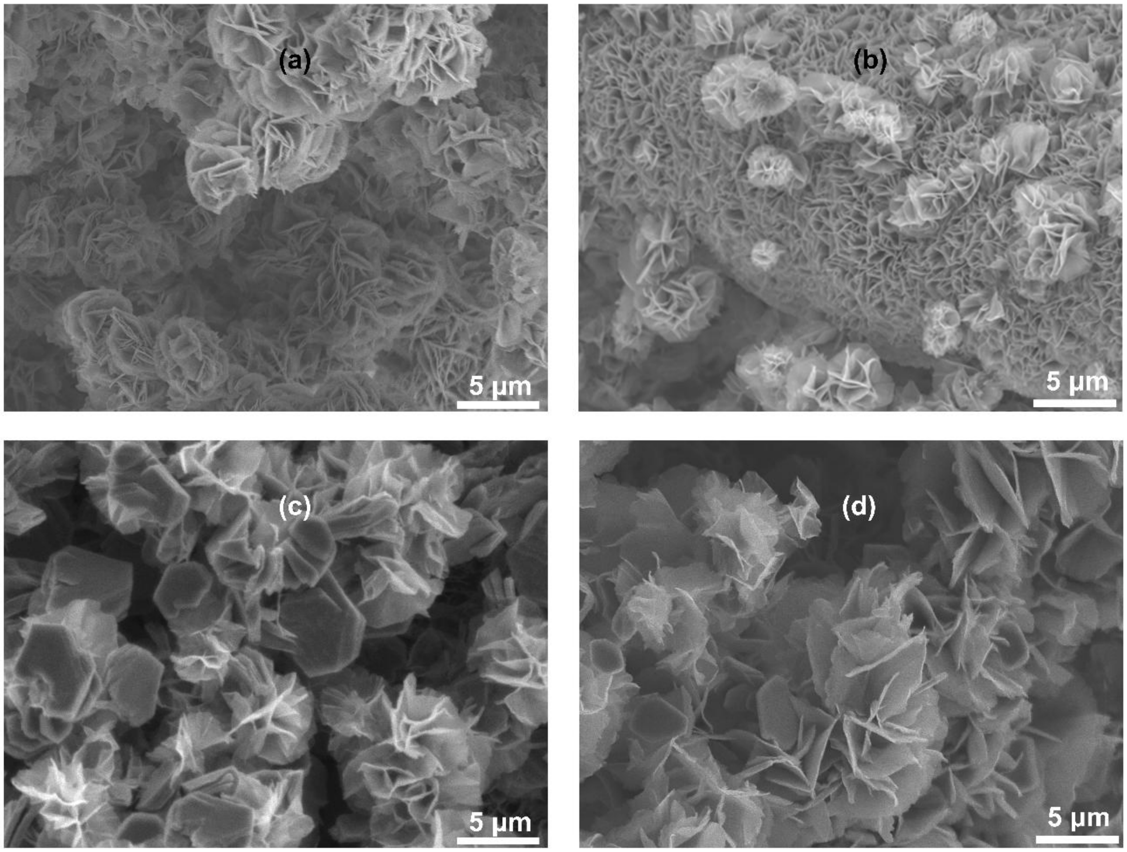

Figure S3. FESEM images of as-synthesized electrodes before CV oxidation in 1 M KOH (a) 1Co@NF and (b) 1NMC@NF, FESEM images of single metal incorporated electrodes after 30 CV oxidation in 1 M KOH (c) 2MC@NFand (d) 2NC@NF.

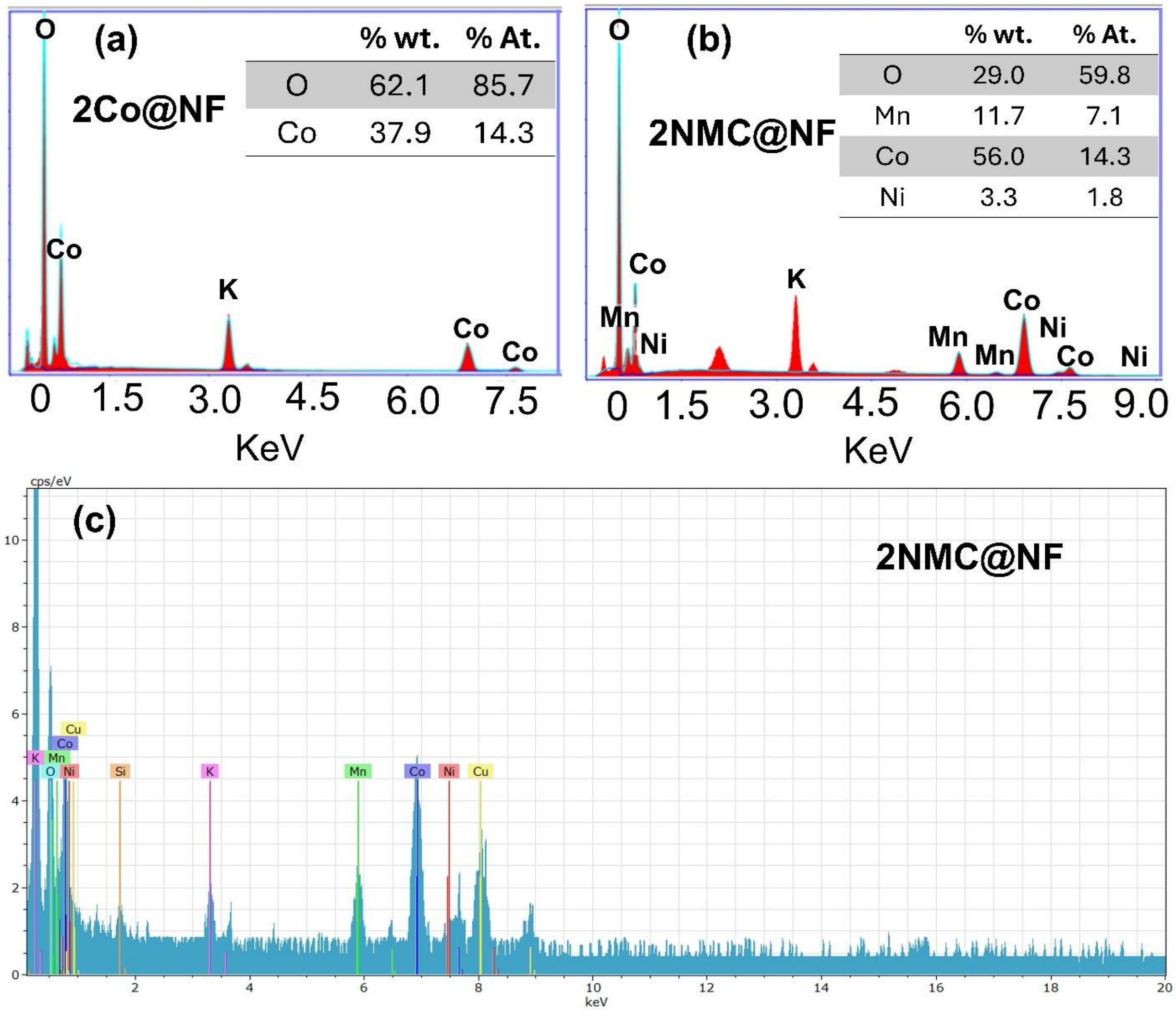

Figure S4. EDS plots and quantification of as-synthesized (a) 2Co@NF and (b) 2NMC@NF, (c) Elemental EDX spectrum of as-synthesized 2NMC@NF. (The Cu peak in Figure S4c results from the Cu lacey carbon film grid used for STEM samples preparation. The K peaks in Figure S4a-c comes from the remnant of 1 M KOH during CV oxidation).

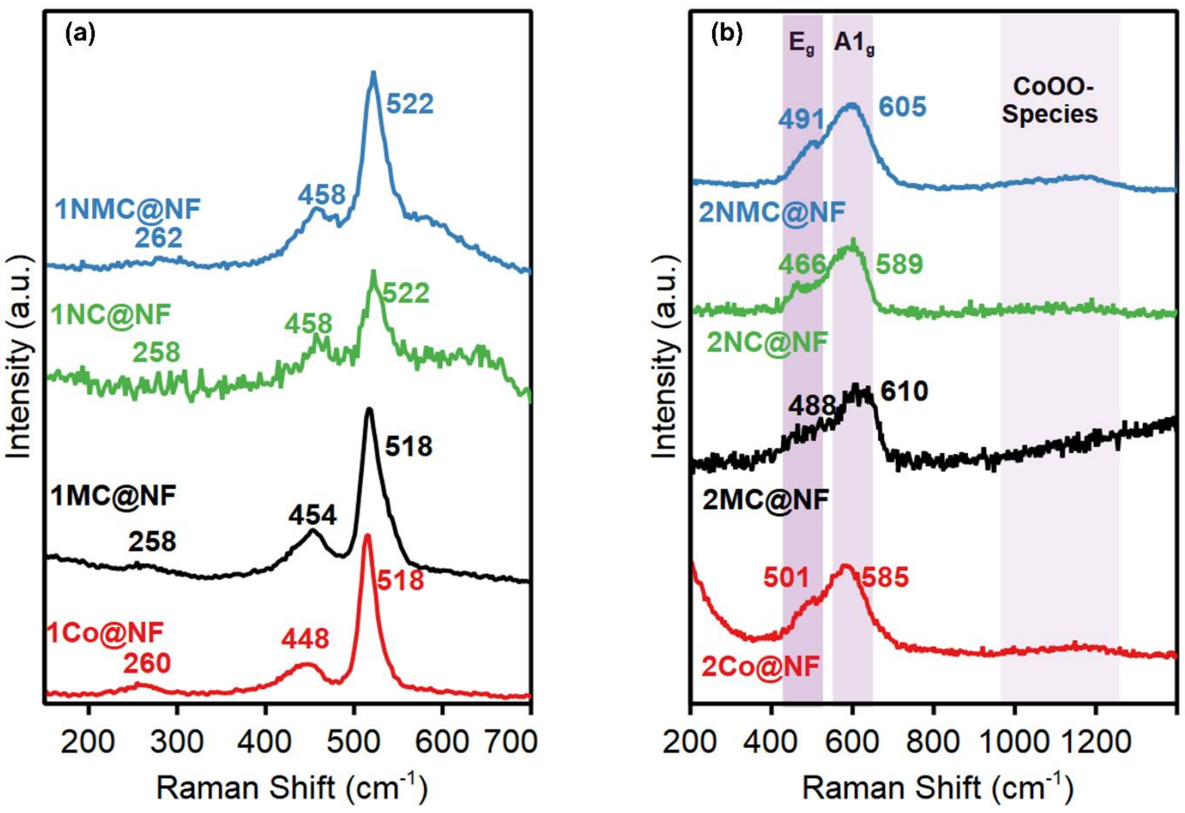

Figure S5: Ex-situ Raman characterizations of (a) as-synthesized 1Co@NF, 1MC@NF, 1NC@NF, and 1NMC@NF and (b) oxidized 2Co@NF, 2MC@NF, 2NC@NF, and 2NMC@NF

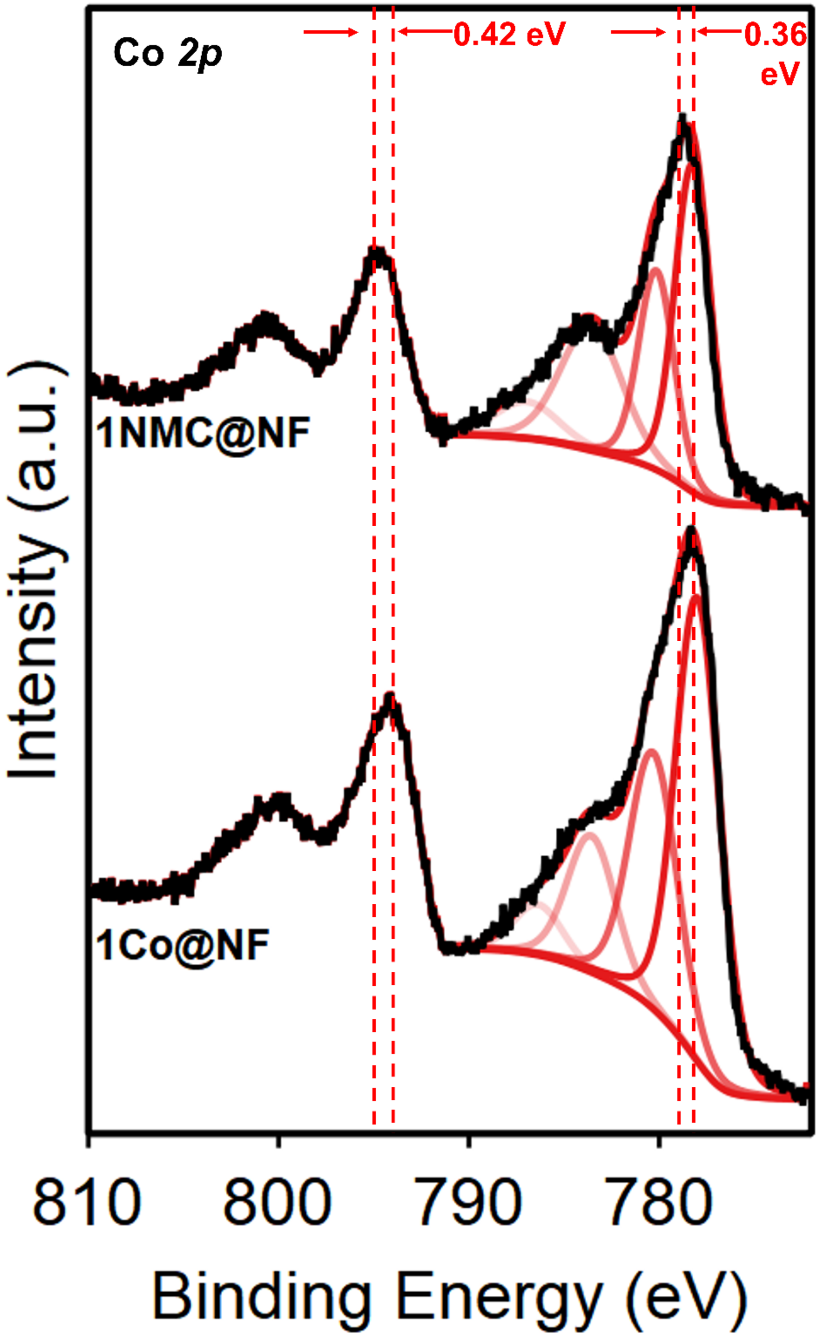

Figure S6. XPS of Co *2p* for as-synthesized 1Co@NF and 1NMC@NF before CV oxidation in 1 M KOH in different resolutions (0.36 and 0.42 eV positive shifts in Co $2p_{3/2}$ and $2p_{1/2}$, respectively, are observed when Ni and Mn are incorporated into 1Co@NF)

Table S2: Potential requirements for BAOR onset and to reach 10 and 50 mA cm^{-2} in all electrocatalysts

Electrocatalyst	Onset Potential (V vs RHE)	Potential @10 mA cm^{-2}	Potential @50 mA cm^{-2}
2Co@NF	1.31 ± 0.04	1.35 ± 0.012	1.47 ± 0.014
2NC@NF	1.24 ± 0.032	1.31 ± 0.006	1.40 ± 0.009
2MC@NF	1.32 ± 0.052	1.35 ± 0.016	1.49 ± 0.017
2NMC@NF	1.25 ± 0.007	1.31 ± 0.009	1.41 ± 0.012
NF	1.35 ± 0.023	1.39 ± 0.004	1.52 ± 0.011

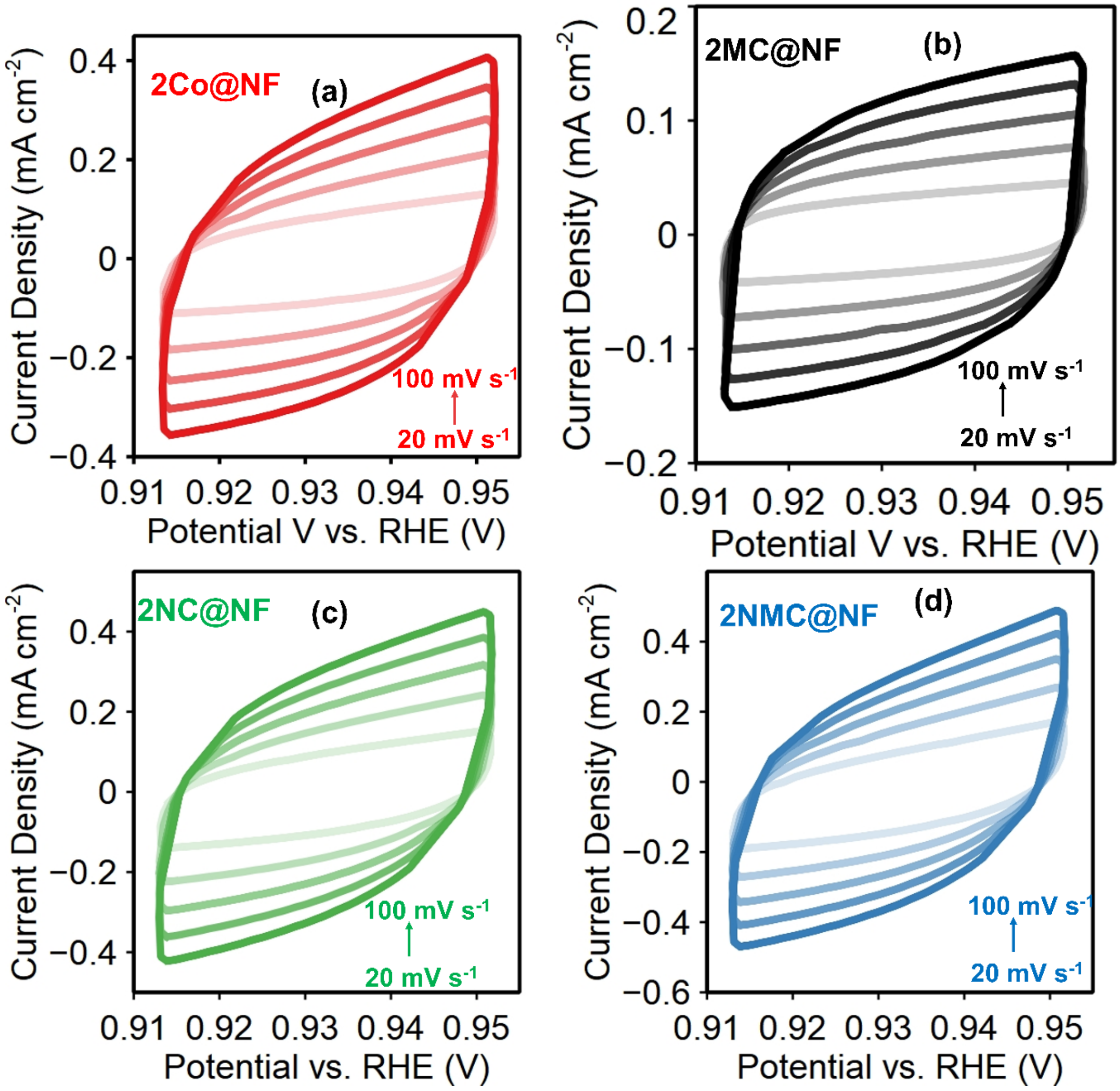

Figure S7: CV at different scan rate in Non-Faradaic regions for ECSA analysis of (a) 2Co@NF, (b)2MC@NF, (c)2NC@NF, and (d)2NMC@NF

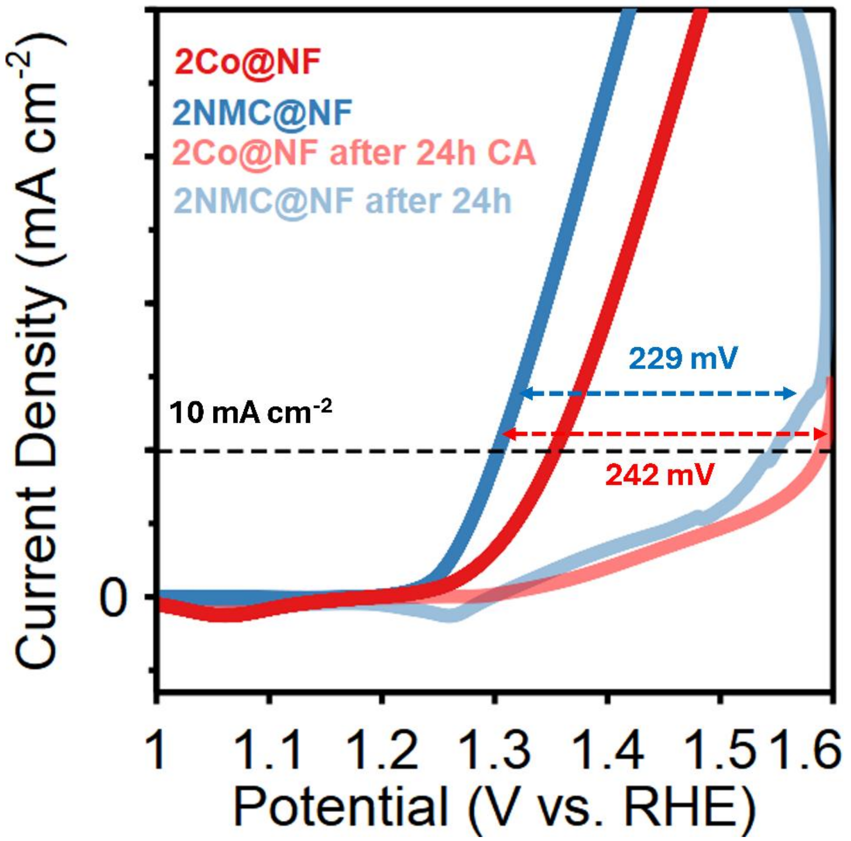

Figure S8: LSV for BAOR in 1 M KOH +0.1 M benzyl alcohol of 2Co@NF and 2NMC@NF after 24 h CA at constant potential of 1.5 V vs RHE without changing electrolyte.

Working Electrode: As-synthesized catalyst; Reference Electrode: Hg/HgO/OH-; Counter Electrode: Carbon fiber paper. Scan rate: 1 mV s^{-1}

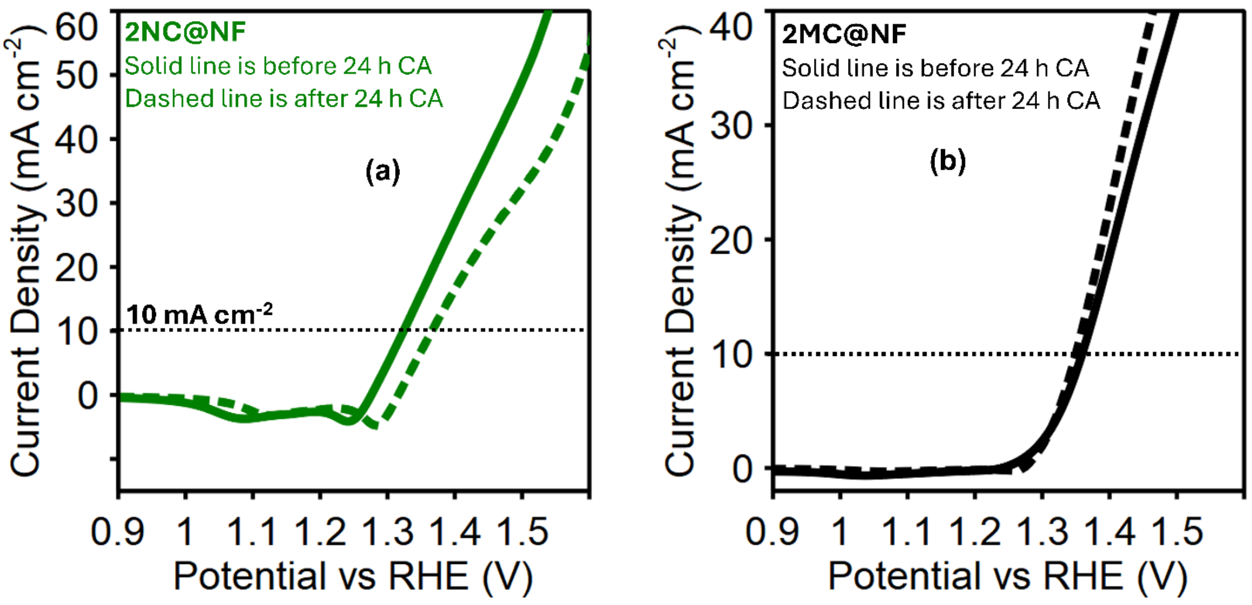

Figure S9: LSV for BAOR in 1 M KOH +0.1 M benzyl alcohol of 2NC@NF and 2MC@NF in a fresh electrolyte after 24 h CA at constant potential of 1.5 V vs RHE.

Working Electrode: As-synthesized catalyst; Reference Electrode: Hg/HgO/OH-; Counter Electrode: Carbon fiber paper. Scan rate: 1 mV s^{-1}

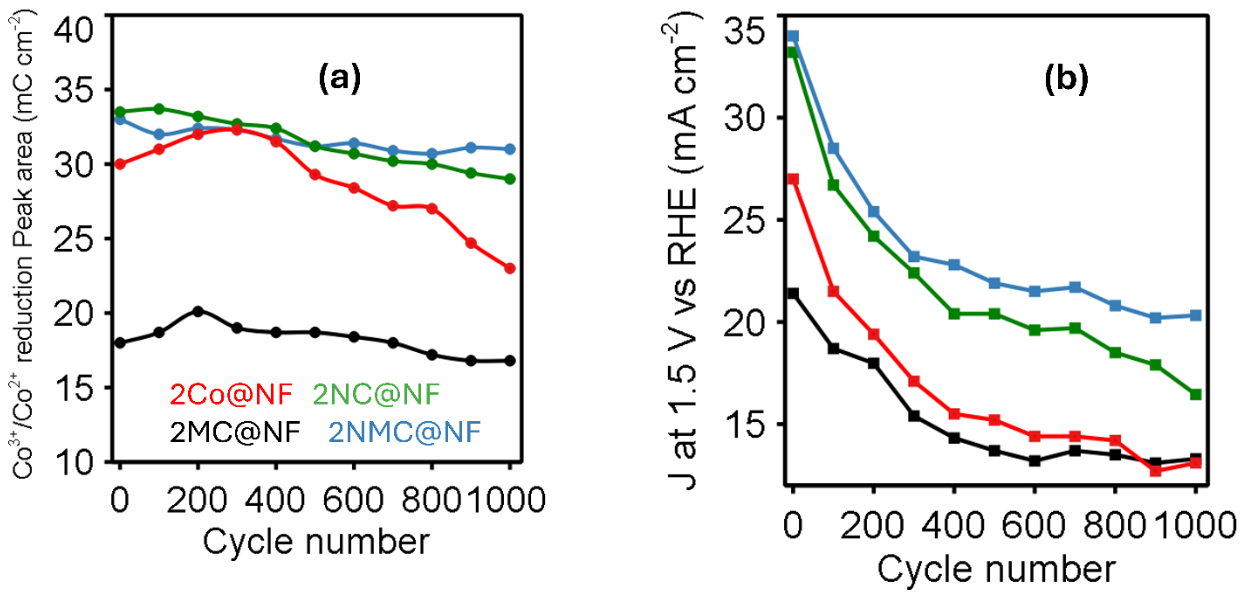

Figure S10 (a) $Co^{2+/3+}$ reduction peak area and (b) current density and BAOR performance from CV cycling.

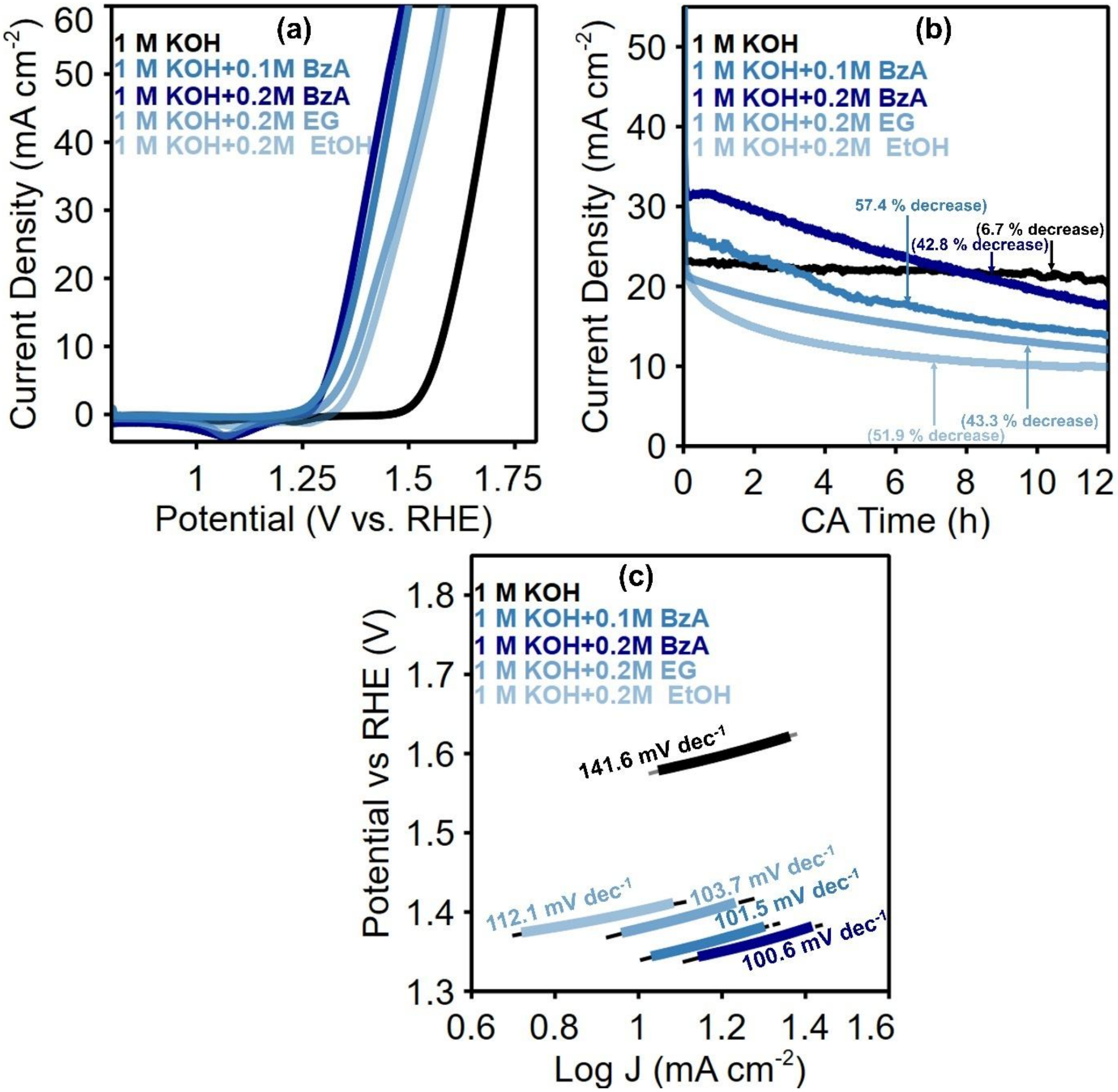

Figure S11: (a) LSV polarization plots (*iR*-corrected) for 2NMC@NF for BAOR, EOR, and EGOR, (b) CA plots of 2NMC@NF for BAOR, EOR, and EGOR at a constant potential of 1.5 V vs RHE for 12 h, and (c) Tafel plots derived from LSV data in (a) for 2NMC@NF for BAOR, EOR, and EGOR

Detail for calculation of Activation energy Temperature-dependent Kinetic study of 2Co@NF and 2NMC@NF

The Arrhenius equation was employed for both catalysts to understand the kinetics. According to the Arrhenius law, the current generated by an electrochemical reaction at a low potential is related to the temperature according to equation (1). [1]

$$\ln j = \ln A - \frac{E_a}{RT} \quad (1),$$

where j is the current density at a specific potential, R is the gas constant (8.314 $Jmol^{-1}K^{-1}$), T is the temperature in K, A is the pre-exponential constant, and E_A is the activation energy. By linearizing, y = ln j, $m = \frac{-E_a}{R}$, $x = \frac{1}{T}$, c = ln A. This implies that plotting ln j versus 1/T, the slope m allows to calculate E_a.

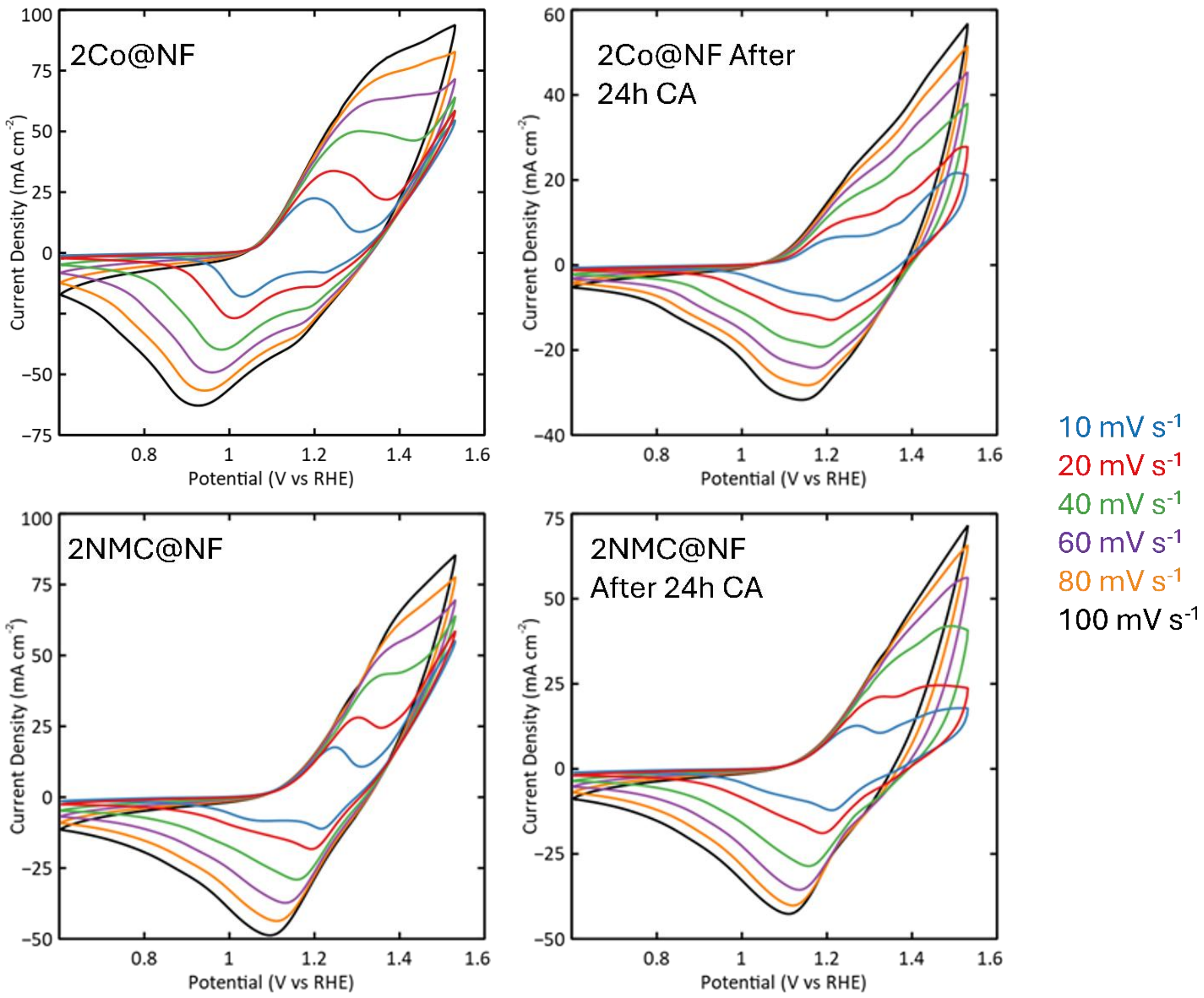

Figure S12: CV plots at different scan rates (10, 20, 40, 60, 80, 100 mV s^{-1}) of 2Co@NF and 2NMC@NF before and after 24h CA in 1 M KOH + 0.1 M benzyl alcohol, constant potential of 1.5 V for Randles Sevcik plot.

Working Electrode: As-synthesized catalyst; Reference Electrode: Hg/HgO/OH-; Counter Electrode: Carbon fiber paper. Scan rate: 1 mV s^{-1}

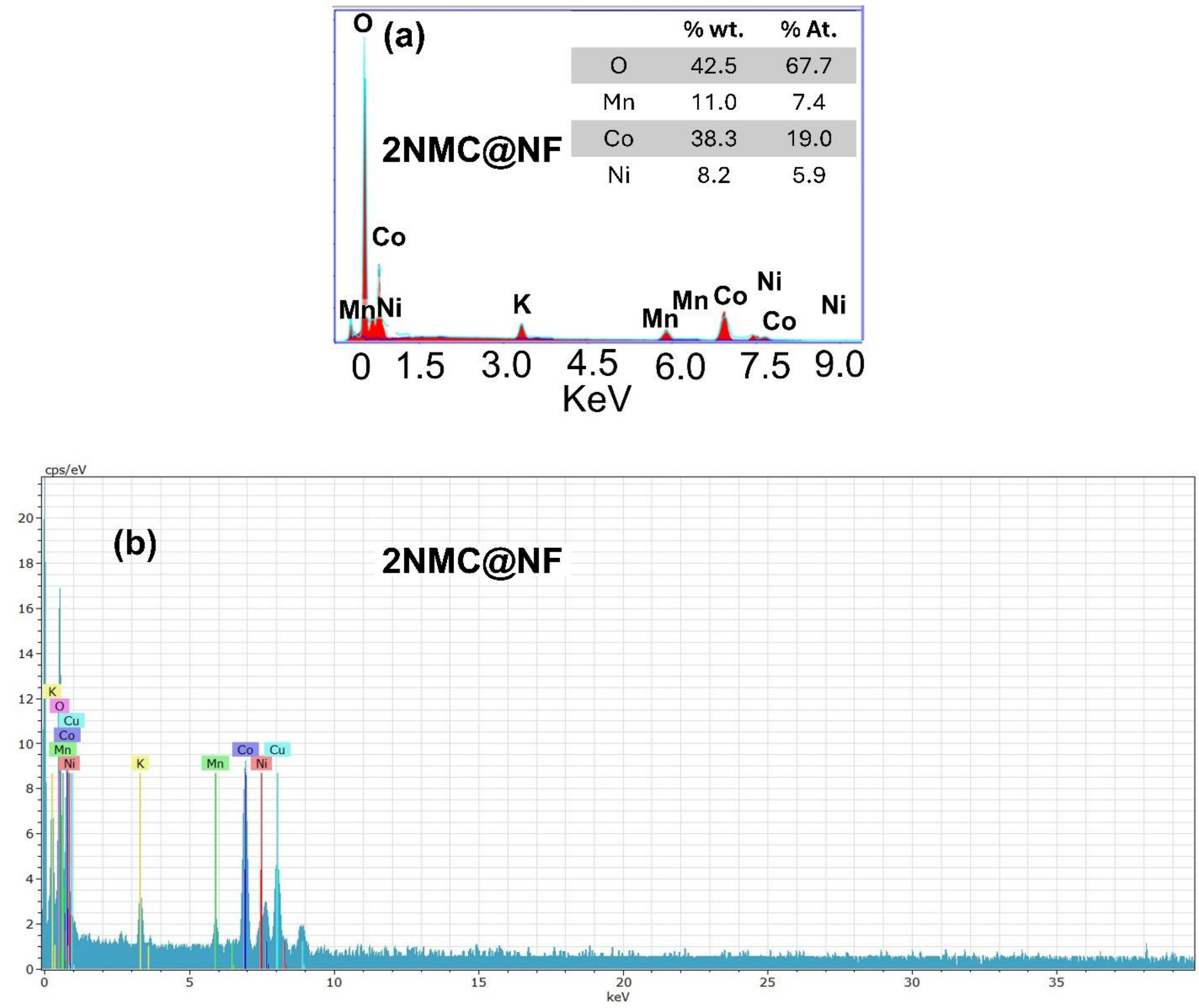

Figure S13. EDS plots and quantification of (a) 2NMC@NF, after 24 h CA stability in 1 M KOH + 0.1 M benzyl alcohol in a constant potential of 1.5 V, (b) Elemental EDX spectrum of as-synthesized 2NMC@NF after 24h CA. (The Cu peak in Figure S4c results from the Cu lacey carbon film grid used for STEM samples preparation. The K peaks in Figure S13a and b come from the remnant of 1 M KOH present in the electrolyte). Reduced Mn intensity is in line with SEM-EDS quantification.

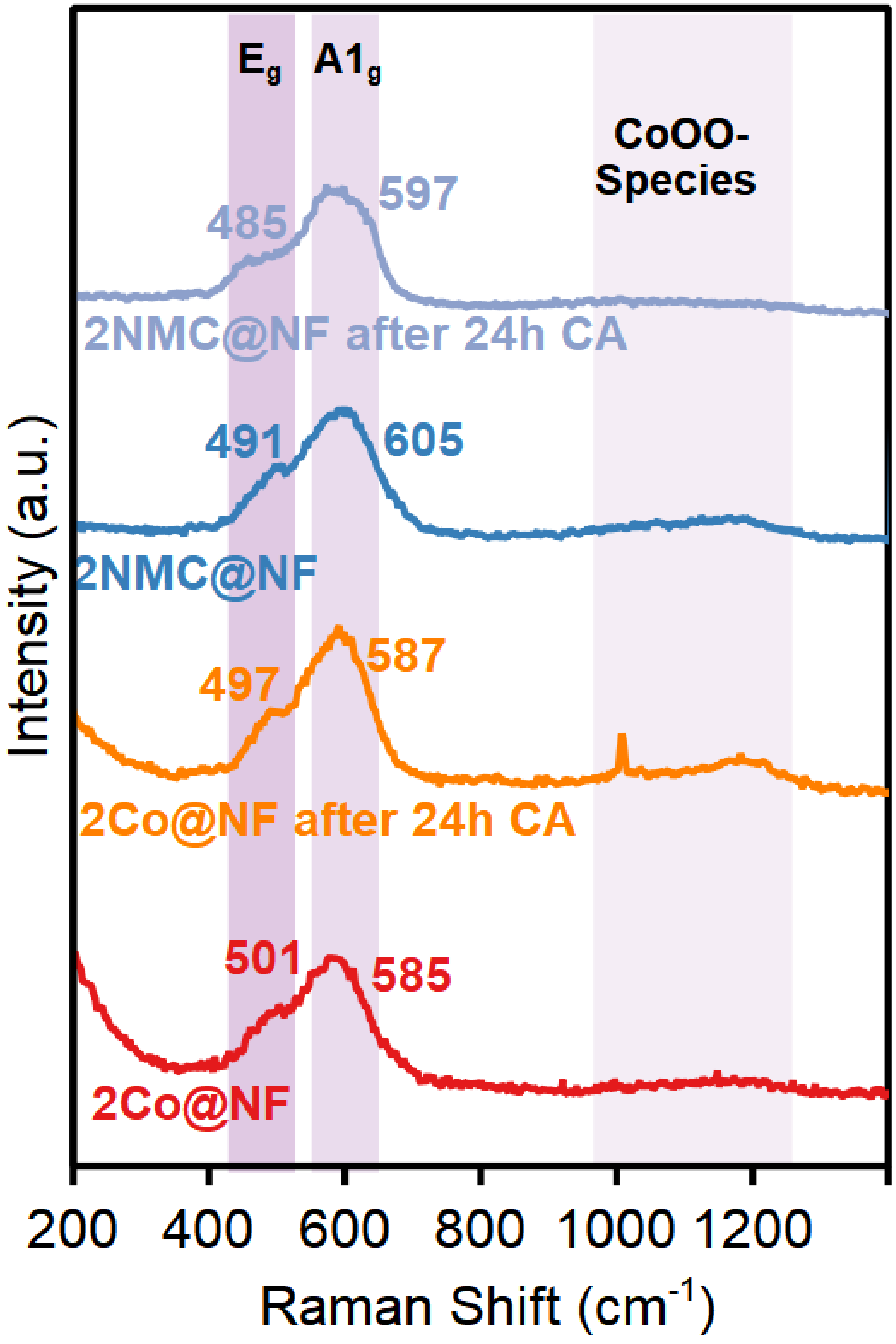

Figure S14: Ex-situ Raman characterizations of 2Co@NF, 2MC@NF, 2NC@NF, and 2NMC@NF after 24h CA

Table S3: XPS surface quantification of 2NMC@NF before and after 24h CA at 1.5 V

Catalysts	Atomic % of Co	Atomic % of Mn	Atomic % of Ni	Ni:Mn:Co
1NMC@NF	61.8±6.7	22.7 ±2.5	17.4±4.3	0.28:0.37:1
2NMC@NF	45.2 ±4.3	27.6±5.9	26.4±6.2	0.58:0.61:1
2NMC@NF after 24 h BAOR	60.1±3.1	24.9±6.4	17.2±8.2	0.29:0.41:1
2NC@NF	73.2±8.2	-	26.8±9.2	
2NC@NF After 24h BAOR	33.2±3.5	-	66.9±7.9	
2MC@NF	54.2±1.9	45.8±5.5	-	
2MC@NF After 24h BAOR	51.3±2.7	49.7±7.1	-	

Table S4: ICP-OES measurents of as-synthesized electrocatalysts, spent electrocatalysts, and spent electrolytes after 24h CA stability at cinstant potential of 1.5 Vvs RHE

Sample	Weight of digested catalyst (mg)	Co(ppm)	Ni(ppm)	Mn(ppm)	Ratio (Co:Ni:Mn)
Fresh electrolyte 1M KOH+0.1M benzyl alcohol	-	0.05	N/A	0.059	1:1:1
Electrolyte used for 2MC@NF	-	0.056	0.039	0.027	1:0.7:0.48
Electrolyte used for 2NC@NF	-	0.045	0.055	0.043	1:1.2:1
Electrolyte used for 2NMC@NF	-	0.022	0.015	N/A	1:0.68:1
Fresh 2MC@NF Electrode	5	10.51	0.81	5.07	1:0.076:0.48
Fresh 2NC@NF Electrode	20	97.76	36.58	0.21	1:0.37:0.002

Fresh 2NMC@NF Electrode	6	15.11	2.54	4.05	1:0.17:0.27
Used 2MC@NF Electrode	6	24.29	0.28	5.79	1:0.012:0.24
Used 2NC@NF Electrode	20	65.47	25.08	0.038	1:0.38:0.00058
Used 2NMC@NF Electrode	6	32.80	5.69	6.23	1:0.17:0.19

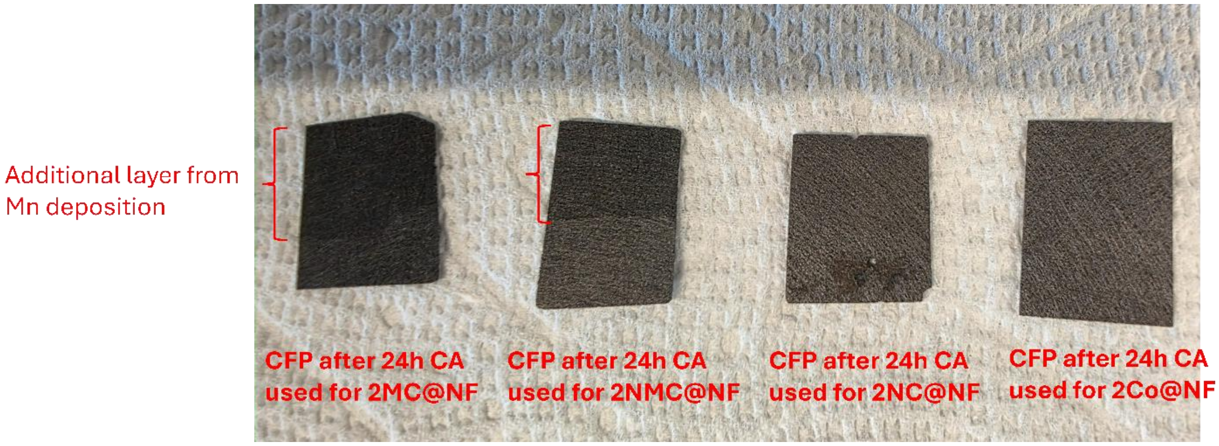

Figure S15: CFP collected after 24h CA stability for all electrocatalysts for Mn re-deposition analysis

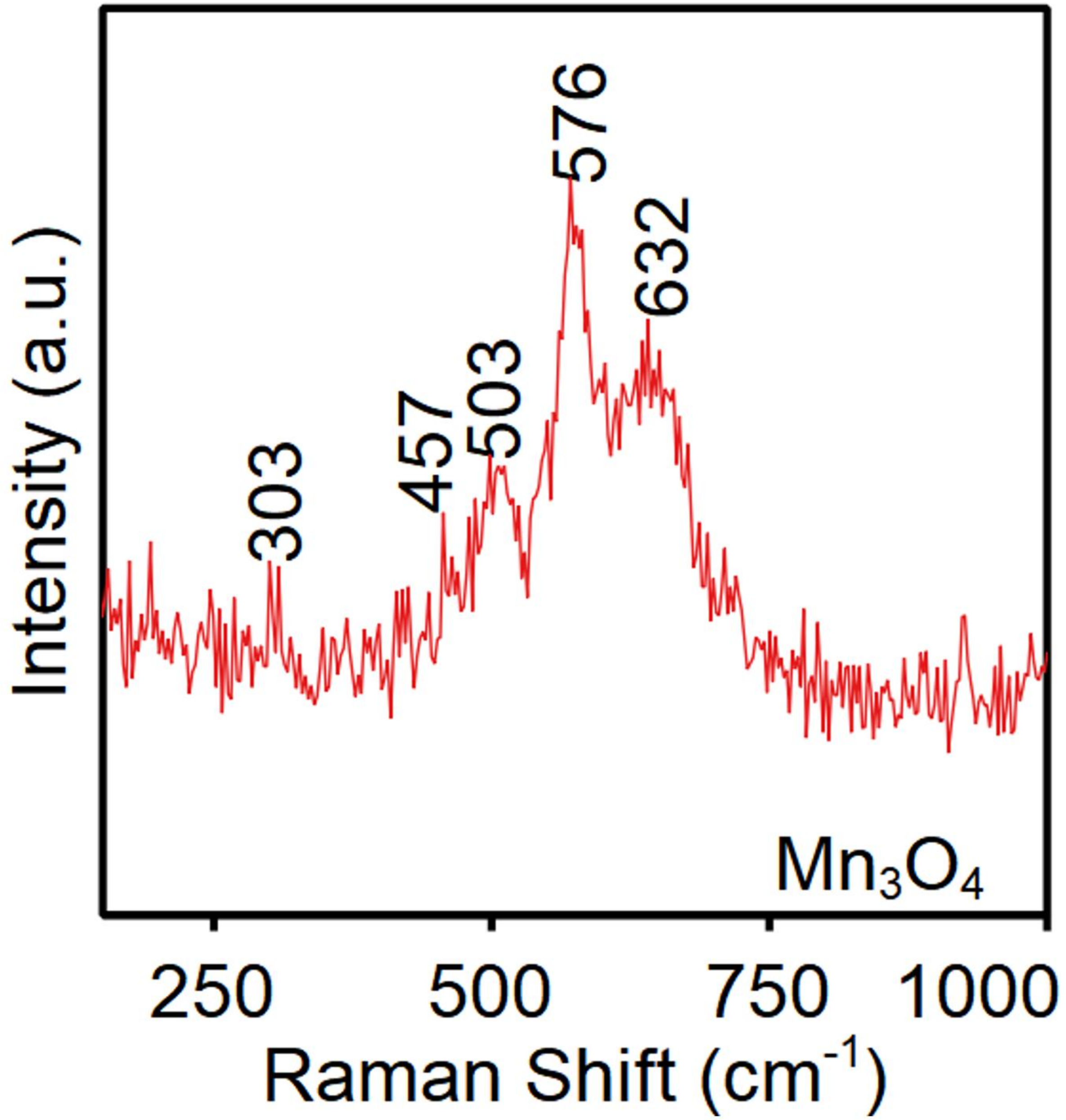

Figure S16: Ex-situ Raman measurement of CFP collected after 24h CA stability for 2NMC@NF electrode for Mn re-deposition analysis.[2]

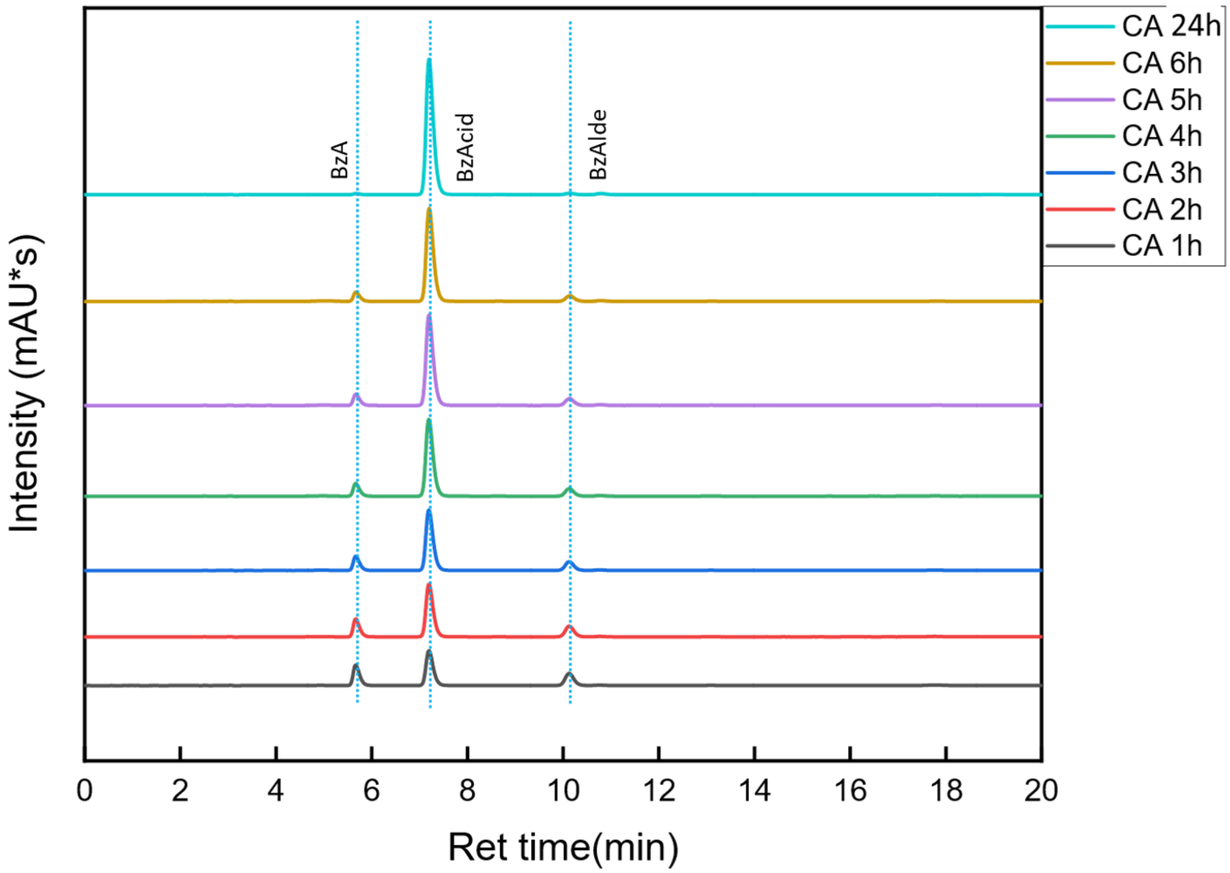

Figure S17. HPLC chromatographs of 1 M KOH + 0.1M benzyl alcohol at different chronoamperometry times. (CA parameters: 1.5V vs RHE for 24 h).

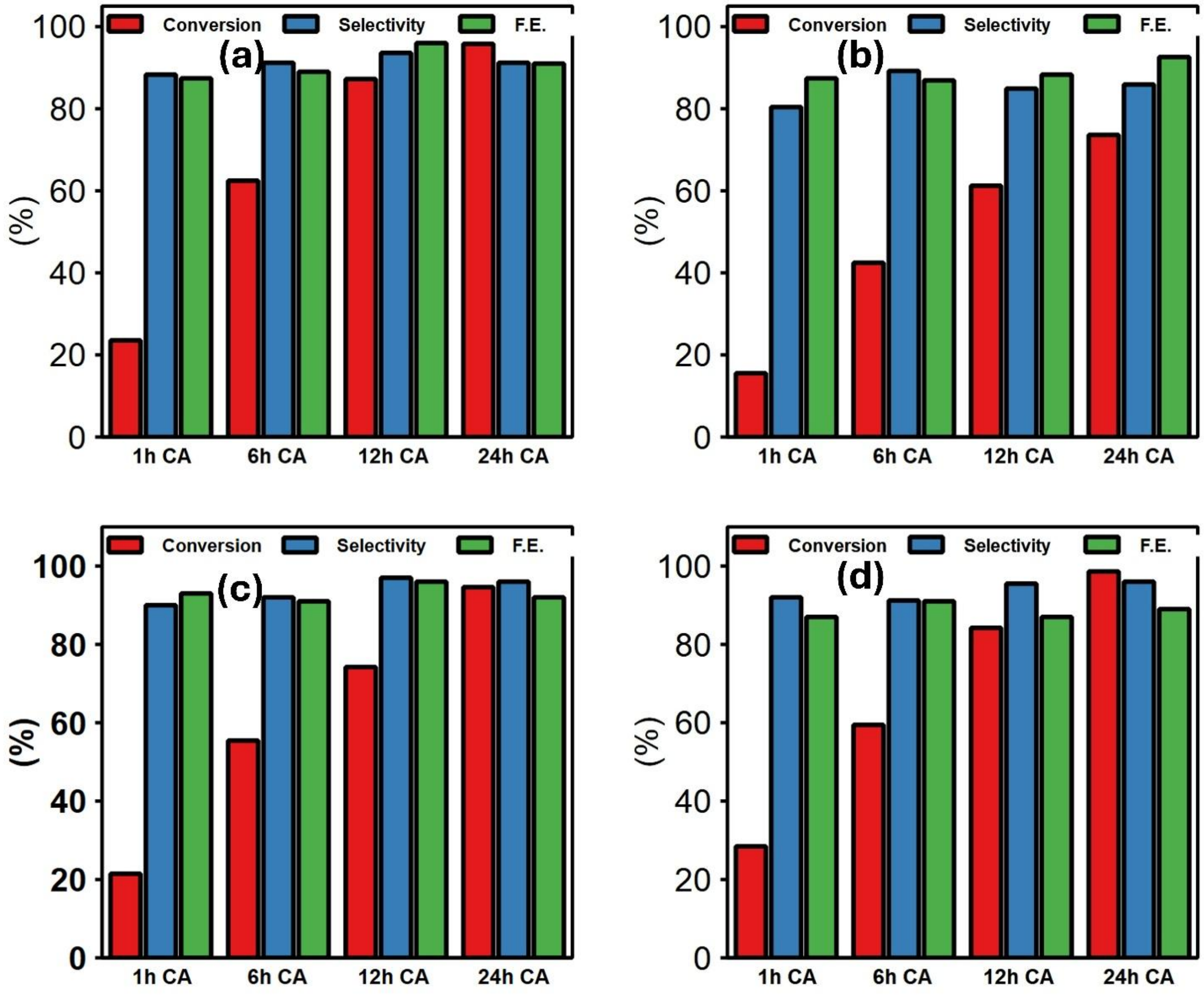

Figure S18: Benzyl alcohol conversion, benzoic acid selectivity, and benzoic acid FE of 2NMC@NF in (a) 1M KOH + 0.05M benzyl alcohol, (b) 1M KOH + 0.2M benzyl alcohol, (c) 1M KOH + 0.1M benzyl alcohol, 30ºC, (d) 1M KOH + 0.1M benzyl alcohol, 40ºC. All measurements were performed by CA for 24h with a constant applied potential of 1.5 V vs RHE.

Table S5. Comparison of the BAOR activity of 2NMC@NF with literature-reported Co-based electrocatalysts

Catalyst	Electrolyte	BAOR activity	Conversion	Selectivity	FE	Ref
$Co_{0.83}Ni_{0.17}$/AC	1 M KOH + 0.01 M BA	≈1.28 V onset potential	100% at 1.425 V	-	96% at 1.425 V	[3]
NC@CuCo_2N_x/CF	1 M KOH + 0.015 M BA	≈1.25 V for 10 mA cm^{-2}	97.3%	98% selectivity towards BAD	81.3%	[4]
NC@CoN_x/CF	1 M KOH + 0.015 M BA	-	86.4%	98% selectivity towards BAD	<70%	[4]
NC@CuCoN_x/CF	1 M KOH + 0.015 M BA	-	90.8%	98% selectivity towards BAD	<70%	[4]
Co(Cu)0.5H_xO_y	1 M KOH with 10 mM BA	≈1.39 V for 10 mA cm^{-2}	95.3% at 0.5 V vs Hg/HgO	98.2% at 0.5 V vs Hg/HgO towards BAC	82% at 0.5 V vs Hg/HgO	[5]
A-CoNi-H/NF	1 M KOH with 0.1M BA	1.35 V for 100 mA cm^{-2}	Around 100% at 1.5 V	Around 100% at 1.5 V towards BAC	93.5% at 1.5 V	[6]
CoNiP/NF	1 M KOH+0.06M BA	1.41 V to 100 mA cm^{-2}	99.5%	97±3% at 1.5 V towards BAC	92±3% at 1.5 V	[7]
Co-Ni-LDH/NF	1 M KOH+0.15M BA	1.3 V Onset	-	-	87 % at 1.4 V	[8]
Co_3Fe_1-LDH	1 M KOH+0.01M BA	1.51 V to 50 mA cm^{-2}	96.8% at 0.8 V vs Ag/AgCl	94.9 % at 0.8 V vs Ag/AgCl towards BAC	71% at 0.8 V vs Ag/AgCl	[9]
$Co(OH)_2$	1 M KOH+0.01 M BA	Higher potential than Co_3Fe_1-LDH	79.6% at 0.8 V vs Ag/AgCl	88.2 % at 0.8 V vs Ag/AgCl towards BAC	-	[9]
Au/CoOOH	1 M KOH+0.1M BA	1.3 V to 340 mA cm^{-2}	99% at 1.3 V	92% at 1.3 V towards BAC	Over 98% at 1.3 V	[10]

2NMC@NF	1 M KOH+0.1M BA @rt	1.41 V to 50 mA cm^{-2}	92.9% ± 3.1 at 1.5 V, 24h	89.7 ± 4.2 % at 1.5 V, 24h towards BAC	91.4 ± 4.7 % at 1.5 V, 24h CA	**This work**
2NMC@NF	1 M KOH+0.1M BA @40ºC	1.38 V to 50 mA cm^{-2}	98.6% at 1.5 V, 24h	96.1% at 1.5 V, 24h towards BAC	89 % at 1.5 V, 24h	**This work**
2NMC@NF	1 M KOH+0.05M BA @rt	1.4 V to 50 mA cm^{-2}	91.2% at 1.5 V, 24h	96.1% at 1.5 V, 24h towards BAC	91 % at 1.5 V, 24h	**This work**
2NMC@NF	1 M KOH+0.2M BA @rt	1.38 V to 50 mA cm^{-2}	97.3 ± 2.1% at 1.6 V, 24h	91.1% at 1.6 V, 24h towards BAC	72.2 % at 1.6 V, 24h	**This work**

Note: BA denotes benzyl alcohol, BAC denotes benzoic acid/benzoate, BAD denotes benzaldehyde, rt denotes room temperature. All potential are reported as V vs RHE except specified.

REFERENCE

(1) Chen, D. P.; Liu, X. C.; Liu, X. D.; Yuan, L.; Zhong, M. L.; Wang, C. Y. Pd Nanoparticles on Self-Doping-Defects Mesoporous Carbon Supports for Highly Active Ethanol Oxidation and Ethylene Glycol Oxidation. *Int. J. Hydrogen Energy* **2021**, *46* (59), 30455–30466. DOI 10.1016/J.IJHYDENE.2021.06.167.

(2) Cho, K. H.; Park, S.; Seo, H.; Choi, S.; Lee, M. Y.; Ko, C.; Nam, K. T. Capturing Manganese Oxide Intermediates in Electrochemical Water Oxidation at Neutral PH by In Situ Raman Spectroscopy. *Angew. Chem. Int. Ed.* **2021**, *60* (9), 4673–4681. DOI 10.1002/ANIE.202014551.

(3) Liu, G.; Zhang, X.; Zhao, C.; Xiong, Q.; Gong, W.; Wang, G.; Zhang, Y.; Zhang, H.; Zhao, H. Electrocatalytic Oxidation of Benzyl Alcohol for Simultaneously Promoting H_2 Evolution by a $Co_{0.83}Ni_{0.17}$/Activated Carbon Electrocatalyst. *New Journal of Chemistry* **2018**, *42* (8), 6381–6388. DOI 10.1039/C8NJ00446C.

(4) Zheng, J.; Chen, X.; Zhong, X.; Li, S.; Liu, T.; Zhuang, G.; Li, X.; Deng, S.; Mei, D.; Wang, J. Hierarchical Porous NC@CuCo Nitride Nanosheet Networks: Highly Efficient Bifunctional Electrocatalyst for Overall Water Splitting and Selective Electrooxidation of Benzyl Alcohol. *Adv. Funct. Mater.* **2017**, *27* (46), 1704169. DOI 10.1002/adfm.201704169.

(5) Huang, L.; Lin, X.; Zhang, K.; Zhang, J.; Wang, C.; Qu, S.; Wang, Y. Extraordinary d–d Hybridization in Co(Cu)0.5OxHy Microcubes Facilitates PhCH2O* –Co(IV) Coupling for Benzyl Alcohol Electrooxidation. *Applied Catalysis B: Environment and Energy* **2024**, *346* (19), 123739. DOI 10.1016/j.apcatb.2024.123739.

(6) Huang, H.; Yu, C.; Han, X.; Huang, H.; Wei, Q.; Guo, W.; Wang, Z.; Qiu, J. Ni, Co Hydroxide Triggers Electrocatalytic Production of High-Purity Benzoic Acid over 400 MA Cm−2. *Energy Environ. Sci.* **2020**, *13* (12), 4990–4999. DOI 10.1039/d0ee02607g.

(7) Guo, S.; Ren, L.; Jia, S.; Zhi, S.; Chang, J.; Ma, X.; Gao, Z. Electrochemical Benzyl Alcohol Oxidation Reaction for the Selective Synthesis of Benzoate and Benzaldehyde Using a Cobalt Nickel Phosphide Catalytic Electrode. *J. Colloid Interface Sci.* **2026**, *24* (12), 140180. DOI 10.1016/j.jcis.2026.140180.

(8) Shilpa, N.; Pandikassala, A.; Krishnaraj, P.; Walko, P. S.; Devi, R. N.; Kurungot, S. Co−Ni Layered Double Hydroxide for the Electrocatalytic Oxidation of Organic Molecules: An Approach to Lowering the Overall Cell Voltage for the Water Splitting Process. *Cite This: ACS Appl. Mater. Interfaces* **2022**, *14*, 16222–16232. DOI 10.1021/acsami.2c00982.

(9) Xu, Y.; Liu, H.; Wu, Y.; Wu, Q.; Li, C.; Wang, X.; Qin, H.; Qin, A.; Wang, L. Fe Electron Center Local Regulation of CoFe-Layered Double Hydroxides Nanosheets for Enhancing the Electrocatalytic Oxidation of Benzyl Alcohol. *ChemNanoMat* **2023**, *9* (12), e202300414. DOI 10.1002/cnma.202300414.

(10) Li, Z.; Yan, Y.; Xu, S.-M.; Zhou, H.; Xu, M.; Ma, L.; Shao, M.; Kong, X.; Wang, B.; Zheng, L.; Duan, H. Alcohols Electrooxidation Coupled with H2 Production at High Current

Densities Promoted by a Cooperative Catalyst. *Nat. Commun.* **2022**, *13* (1), 147. DOI 10.1038/s41467-021-27806-3.
